# Integrative Chemical and Omics Analyses Provide Insights into Pentlandite Bioleaching by *Acidithiobacillus ferriphilus* WGS1

**DOI:** 10.3390/ijms27135762

**Published:** 2026-06-26

**Authors:** Yan Tong, Yuandong Liu

**Affiliations:** 1 School of Minerals Processing and Bioengineering, Central South University, Changsha 410083, China; yantong@csu.edu.cn; 2 Key Laboratory of Biohydrometallurgy of Ministry of Education, Changsha 410083, China

**Keywords:** pentlandite, bioleaching, *Acidithiobacillus ferriphilus*, nickel resistance, iron–sulfur oxidation, transcriptomics

## Abstract

Pentlandite bioleaching offers a potentially low-energy route for nickel recovery from low-grade sulfide resources, but increasing pulp density may constrain acidophilic microorganisms through metal accumulation, mineral buffering, mass-transfer limitation, and surface-product deposition. This study evaluated pentlandite bioleaching by the nickel-resistant *Acidithiobacillus ferriphilus* WGS1 at pulp densities of 1%, 5%, and 10% (*w*/*v*). Leaching performance and associated interfacial and cellular responses were examined using solution chemistry, mineral and surface characterization, electrochemical measurements under 40 g/L Ni^2+^, and genome-guided transcriptomics. After 30 days at 35 °C, Ni leaching efficiencies reached 99.2%, 97.1%, and 95.7% at 1%, 5%, and 10% pulp densities, respectively, compared with 27.2%, 14.2%, and 0.76% in the corresponding sterile controls. The inoculated systems maintained lower pH and higher ORP than the sterile controls, while the residues showed pentlandite alteration, Ni depletion, secondary Fe-bearing phase formation, and changes in surface sulfur speciation. Under 40 g/L Ni^2+^, the WGS1-containing system showed a lower charge-transfer resistance and a higher corrosion current density than the abiotic system. Transcriptomic comparison between the 10% and 1% pulp-density groups identified 640 differentially expressed genes and highlighted candidate responses associated with Ni homeostasis, Fe/S oxidation, respiratory electron transfer, and energy conservation. Integration of the physicochemical, mineralogical, electrochemical, and transcriptomic results supports a literature-informed working model for WGS1-associated pentlandite bioleaching under high-pulp-density conditions.

## 1. Introduction

Nickel is essential for stainless steels, corrosion-resistant alloys, superalloys, and nickel-rich battery materials, and demand for sustainable nickel resources is increasing [1]. In magmatic nickel sulfide deposits, pentlandite ((Fe,Ni)_9_S_8_) is the principal nickel-bearing sulfide mineral, and its dissolution behavior directly controls Ni release and Fe/S transformation. As high-grade sulfide resources are progressively depleted, bioleaching offers a mild and potentially greener route for utilizing low-grade nickel sulfide resources [2,3,4].

The bioleaching of metal sulfides is mainly driven by microbially mediated iron and sulfur oxidation. Iron-oxidizing microorganisms regenerate ferric ions (Fe^3+^), which chemically attack sulfide mineral surfaces, whereas sulfur-oxidizing microorganisms convert reduced inorganic sulfur compounds into sulfate and regenerate acidity. Together, these reactions maintain low pH and high oxidation–reduction potential (ORP), thereby sustaining mineral dissolution [3]. During pentlandite bioleaching, H^+^, Fe^3+^, and attached microorganisms may jointly promote the cleavage and oxidation of Ni-S and Fe-S bonds. However, sulfur-rich layers, jarosite, and iron oxyhydroxides may accumulate on mineral surfaces and contribute to passivation [5]. Previous studies have demonstrated the feasibility of pentlandite bioleaching, but process efficiency can be constrained by acid consumption, secondary-mineral precipitation, sulfur passivation, and mass-transfer limitations [5,6,7,8].

A further challenge is the accumulation of Ni^2+^ during mineral dissolution. Excess Ni^2+^ can disrupt cellular metal homeostasis, interfere with metalloproteins and Fe-S cluster-dependent metabolism, impair respiratory function, and induce oxidative stress [9,10]. These effects may become more pronounced at high pulp densities because of increased metal-ion loads, mineral buffering, and mass-transfer limitations. Although *Acidithiobacillus* species are widely used in biomining, the mechanisms by which nickel-resistant strains sustain pentlandite dissolution under combined high-Ni^2+^ and high-pulp-density stress remain insufficiently understood. Integrating mineral interfacial characterization with genome-guided transcriptomic analysis may provide further insight into the cellular responses potentially associated with this process.

Here, we evaluated pentlandite bioleaching by the nickel-resistant acidophile *A. ferriphilus* WGS1 at pulp densities of 1%, 5%, and 10% (*w*/*v*). Solution chemistry, mineral-surface characterization, and electrochemical measurements were used to characterize leaching performance and associated interfacial changes. Genome-guided transcriptomics was further used to identify candidate cellular responses associated with the high-pulp-density condition. Integration of these datasets yielded a literature-informed working model linking the observed leaching and interfacial phenotypes to candidate responses involving Ni homeostasis, Fe/S oxidation, and respiratory electron transfer, thereby providing a framework for subsequent functional investigation.

## 2. Results and Discussion

### 2.1. Monitoring of Solution Behavior in the Leaching Process

To investigate the bioleaching performance of *A. ferriphilus* WGS1 toward pentlandite at different pulp densities, three solution systems were established: (i) a bioleaching group with 1% pulp density (Bio-1%) and its sterile control (Abio-1%); (ii) a bioleaching group with 5% pulp density (Bio-5%) and its sterile control (Abio-5%); and (iii) a bioleaching group with 10% pulp density (Bio-10%) and its sterile control (Abio-10%).

**Figure 1:**
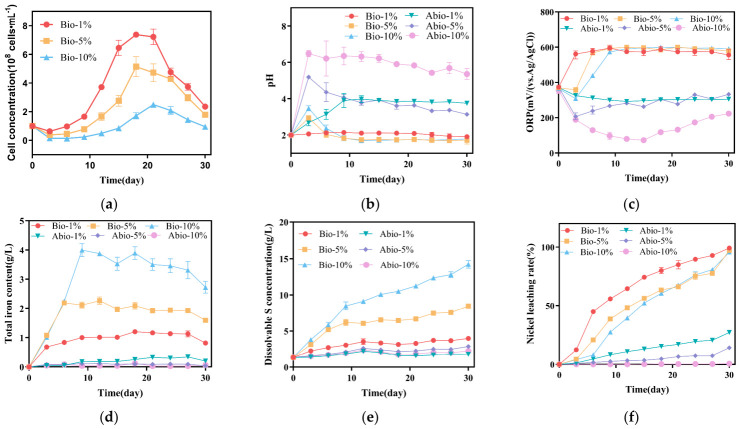
Variation in parameters during the bioleaching of pentlandite by *A. ferriphilus* WGS1 at different pulp densities. (**a**) Cell concentration; (**b**) pH value; (**c**) oxidation–reduction potential (ORP); (**d**) total iron concentration; (**e**) dissolvable S concentration; (**f**) nickel leaching rate. Bio- and Abio- represent inoculated and sterile control groups, respectively.

As shown in Figure 1a, the planktonic cell concentrations in the three bioleaching systems showed an initial increase followed by a decline. The peak planktonic cell concentrations in the Bio-1%, Bio-5%, and Bio-10% groups were approximately 7.4 × 10^8^, 5.0 × 10^8^, and 2.5 × 10^8^ cells/mL, respectively, and the time required to reach the peak was delayed as pulp density increased. Because only planktonic cells were counted, these data reflect suspended-cell abundance rather than total biomass. The lower planktonic cell concentration at higher pulp density may reflect Ni/solid-loading stress and/or redistribution of cells between the liquid phase and mineral surfaces [11,12].

The pH profiles of the inoculated systems varied with pulp density (Figure 1b). During the first 3 days, the pH of Bio-1% remained close to the initial value, whereas the pH values of Bio-5% and Bio-10% increased to approximately 2.9 and 3.5, respectively. Thereafter, Bio-1% fluctuated within a narrow range of 1.87–2.13 throughout the leaching period. In Bio-5%, the pH decreased after the initial increase and stabilized after day 9 at 1.62–1.86, whereas in Bio-10%, it stabilized after day 12 at 1.69–1.80. These results show that the inoculated systems remained acidic or returned to acidic conditions after the initial buffering stage, particularly at 5% and 10% pulp densities.

In contrast, the sterile controls showed higher pH values than the inoculated systems (Figure 1b). In Abio-1%, the pH increased to approximately 3.9 on day 9 and then showed no substantial change, remaining around 3.75–3.90 until day 30. In Abio-5%, the pH increased to approximately 5.2 on day 3 and then gradually declined to 3.1. In Abio-10%, the pH reached approximately 6.6 on day 6 and remained high at 5.24–5.48 until the end of the experiment. The decrease observed in Abio-5% was not accompanied by the high ORP, elevated dissolved Fe/S levels, or high Ni extraction observed in the inoculated systems, suggesting that it was more likely caused by abiotic mineral–solution reactions rather than microbial contamination. These higher pH values coincided with very low nickel leaching efficiencies in the sterile controls, especially in the high-pulp-density system.

ORP was monitored to compare the oxidizing state of the leaching solutions (Figure 1c). In the inoculated groups, ORP increased rapidly at all three pulp densities during the initial leaching stage. In Bio-1%, the ORP increased rapidly during the first 3 days, reaching 534–579 mV (vs. Ag/AgCl) among the triplicate experiments. Thereafter, the ORP remained at a high oxidizing level, generally within the range of 552–611 mV during the subsequent leaching period. The ORP values in Bio-5% and Bio-10% also reached plateau levels of approximately 570 and 575 mV on days 6 and 9, respectively, showing that the inoculated systems maintained high ORP values during leaching. In the sterile controls, the ORP values of Abio-1% and Abio-5% initially decreased to 270–330 mV and then slowly recovered. By contrast, the ORP of Abio-10% dropped to 70–99 mV among the triplicate experiments on day 9 and recovered to only about 220 mV by day 30. The ORP values in the sterile controls, particularly in Abio-10%, were much lower than those in the inoculated systems, and the corresponding Ni leaching efficiencies were also low.

The variation in total dissolved iron concentration was compared among the different leaching systems (Figure 1d). In the inoculated groups, dissolved iron increased during the first 9–12 days at all pulp densities. The peak iron concentrations ranged from 3.6 to 4.3 g/L in Bio-10%, from 2.1 to 2.4 g/L in Bio-5%, and from 1.0 to 1.2 g/L in Bio-1%. In contrast, iron concentrations in all sterile controls remained low throughout the experiment, generally below 0.30 g/L, with maximum values not exceeding 0.39 g/L. In the middle and late stages of leaching, particularly after day 15, iron concentrations in the inoculated groups gradually decreased.

The dissolved S concentration was monitored to compare sulfur release among the different leaching systems (Figure 1e). At the initial time point, soluble sulfur concentrations in all groups ranged from 1.31 to 1.38 g/L and then increased during leaching, with greater increases in the inoculated groups than in the sterile controls. By day 30, soluble sulfur concentrations ranged from 14.1 to 14.5 g/L in Bio-10%, from 8.4 to 8.5 g/L in Bio-5%, and from 3.9 to 4.0 g/L in Bio-1%, whereas the corresponding sterile controls reached only approximately 2.2, 2.8, and 1.7 g/L. Thus, the inoculated systems showed greater accumulation of dissolved sulfur than the sterile controls.

The nickel leaching efficiency results showed clear differences between the inoculated groups and the sterile controls (Figure 1f). During the 30-day leaching period, nickel leaching efficiency in the inoculated groups increased continuously, whereas the sterile controls remained at very low levels and increased only slowly. By day 30, the nickel leaching efficiencies in Bio-1%, Bio-5%, and Bio-10% reached approximately 99.2%, 97.1%, and 95.7%, respectively, all remaining above 95%. In contrast, the corresponding values in Abio-1%, Abio-5%, and Abio-10% were only approximately 27.2%, 14.2%, and 0.76%. Compared with the sterile controls, the inoculated systems increased nickel leaching efficiency by approximately 3.6-fold at low pulp density (1%) and by approximately 126-fold at high pulp density (10%), showing much higher nickel extraction in the presence of WGS1 under the tested conditions.

### 2.2. Analysis of Leaching Residues

#### 2.2.1. SEM-EDS Analysis of Leaching Residue

**Figure 2:**
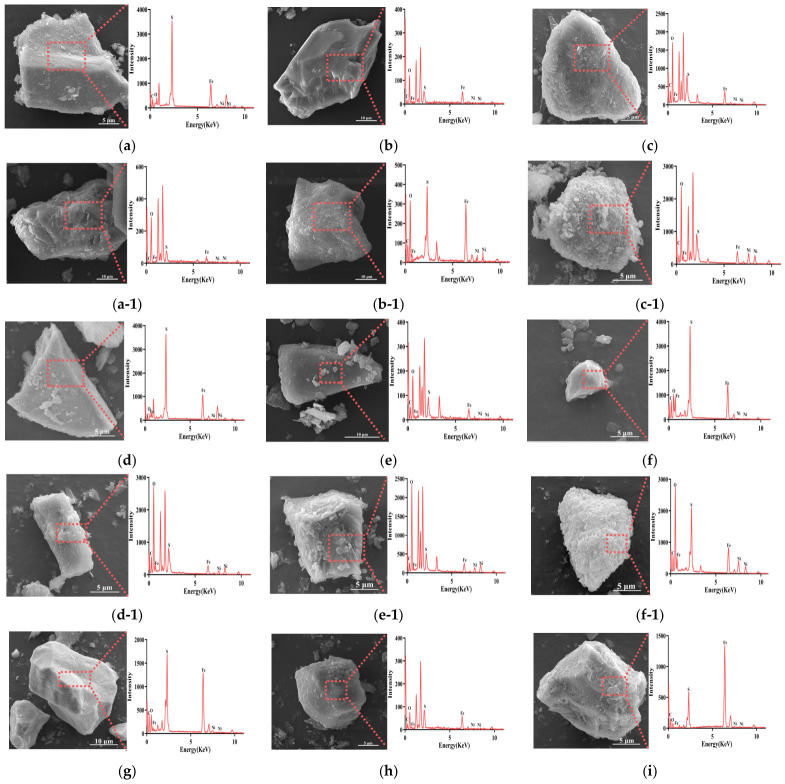

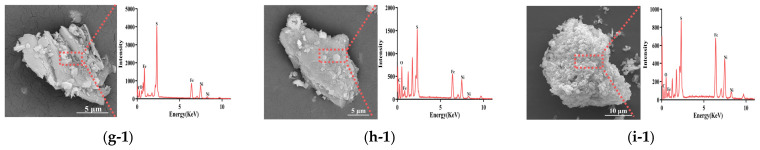
Scanning electron microscope (SEM) images and energy-dispersive X-ray spectroscopy (EDS) spectra of pentlandite residues under different leaching conditions. (**a**–**c**) Abiotic control groups at 1% pulp density on days 10, 20, and 30, respectively; (**a-1**–**c-1**) corresponding bioleaching groups at 1% pulp density; (**d**–**f**) abiotic control groups at 5% pulp density on days 10, 20, and 30, respectively; (**d-1**–**f-1**) corresponding bioleaching groups at 5% pulp density; (**g**–**i**) abiotic control groups at 10% pulp density on days 10, 20, and 30, respectively; (**g-1**–**i-1**) corresponding bioleaching groups at 10% pulp density. XRD analysis of leaching residue. The red dashed boxes indicate the areas selected for EDS analysis.

To compare surface morphological and elemental changes during leaching, SEM-EDS characterization was performed on residues collected from three pulp-density groups at the early (10 d), middle (20 d), and final (30 d) stages of leaching (Figure 2). In the sterile control groups (Figure 2a–i), the mineral particles showed highly similar surface morphologies across all pulp densities throughout the leaching period. The particles retained clear outlines, sharp edges, and smooth surfaces, with no obvious corrosion pits or attached dissolution products, closely resembling the raw ore. This observation was consistent with the persistently low leaching efficiencies (≤27.2%) observed in the sterile controls. The intensities and relative ratios of the characteristic S, Fe, and Ni peaks in the EDS spectra also changed little from 10 to 30 d, suggesting only limited surface alteration in the sterile systems.

In contrast, the surfaces of the inoculated residues (Figure 2(a-1)–(i-1)) exhibited pronounced time-dependent changes. After 10 d of leaching, the mineral surfaces had already become noticeably roughened, with localized signs of microscopic erosion, showing more evident surface alteration than in the sterile controls. By 20 d, corrosion pits were more developed, and granular secondary deposits appeared locally. The Fe, Ni, and S peaks in the EDS spectra also changed in intensity and profile, indicating changes in surface elemental composition during leaching. At 30 d, the inoculated group with 1% pulp density (c-1) showed the most severe particle fragmentation and surface dissolution, with the Ni peak nearly disappearing, in agreement with its nickel leaching efficiency of 99.2%. The 5% group (f-1) displayed similar deep erosion features. In the 10% pulp-density group (i-1), particle erosion was slightly less extensive; however, the Ni peak was still markedly weakened, consistent with the 95.7% leaching efficiency of the Bio-10% group. Overall, the morphological evolution and EDS elemental changes were consistent with progressive surface alteration and nickel depletion in the inoculated residues.

#### 2.2.2. XRD Analysis of Leaching Residue

**Figure 3:**
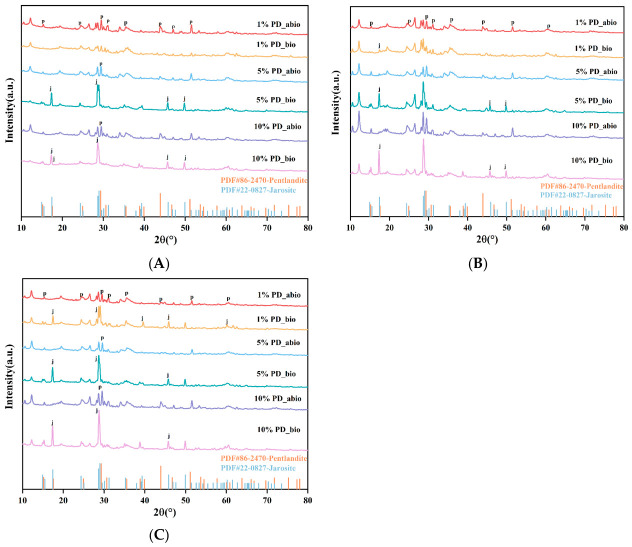
X-ray diffraction (XRD) patterns of the pentlandite leaching residues. Residues from abiotic controls (sterile) and *A. ferriphilus* WGS1 bioleaching groups under different pulp densities (1%, 5%, and 10% PD) at (**A**) 10 days, (**B**) 20 days, and (**C**) 30 days. The labels ‘p’ and ‘j’ indicate the characteristic peaks of pentlandite and jarosite, respectively.

To examine mineral-phase changes in the leaching residues, XRD analysis was performed on residues from each group at the early (10 d), middle (20 d), and late (30 d) stages of leaching (Figure 3). By comparison with standard reference cards (PDF#86-2470 for pentlandite and PDF#22-0827 for potassium jarosite), two main mineral phases were assigned. The characteristic peaks of pentlandite (marked as p) occurred at 2θ = 29.32°, 43.91°, and 51.15°, whereas the characteristic peaks of potassium jarosite (marked as j) appeared at 2θ = 28.97°, 28.68°, and 17.41°.

Throughout the leaching period, the diffraction patterns of all sterile control groups were dominated by pentlandite peaks. These peaks remained sharp, and their intensities showed no substantial temporal variation, suggesting limited mineral-phase change in the sterile residues. Jarosite peaks were absent or only weak in the sterile groups.

In the inoculated groups, the diffraction patterns showed time-dependent changes in pentlandite and jarosite signals. At 10 d, pentlandite remained the dominant crystalline phase, whereas jarosite peaks were not yet prominent. However, some pentlandite peaks showed lower relative intensities than those in the sterile controls, suggesting early alteration of the pentlandite residues in the inoculated systems. By 20 d, the jarosite peaks at 2θ ≈ 28.68° and 28.97° became more evident in the 5% and 10% pulp-density inoculated groups, while the relative intensities of pentlandite peaks decreased. These changes indicate the accumulation of jarosite-type secondary phases and a progressive reduction in pentlandite signals during leaching.

At 30 d, the differences between the inoculated and sterile residues became more pronounced. Within the main jarosite peak region over the 2θ range of 28.6–29.1°, the peak intensity of the 10% PD_bio group was approximately 14,843 a.u., whereas that of the 10% PD_abio group was approximately 6734 a.u. at the same time point. This result shows a stronger jarosite signal in the inoculated high-pulp-density residue but does not by itself quantify Fe^2+^/Fe^3+^ oxidation rates. Meanwhile, the characteristic pentlandite peaks in all inoculated groups were weaker than those in the corresponding sterile controls. This decrease was most evident in the 1% PD_bio group at 30 d, in which the pentlandite peaks had nearly disappeared, consistent with extensive pentlandite dissolution and the high Ni leaching efficiency observed in this group. Overall, the coexistence of weakened pentlandite peaks and enhanced jarosite peaks is consistent with progressive pentlandite dissolution and secondary iron-phase formation in the inoculated residues.

#### 2.2.3. FTIR Spectral Analysis of Leaching Residue

**Figure 4:**
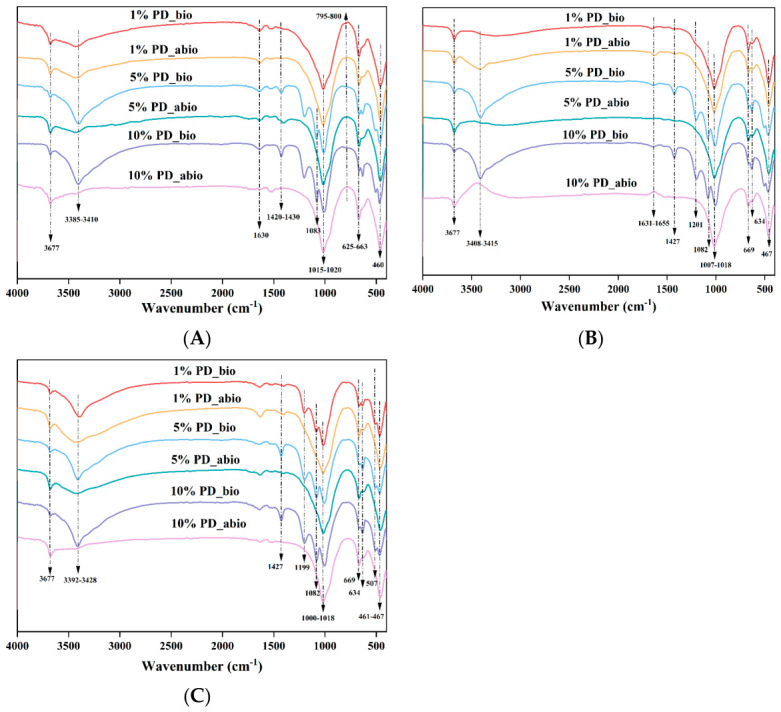
Fourier transform infrared (FTIR) spectra of the pentlandite leaching residues. Spectra of abiotic controls (sterile) and *A. ferriphilus* WGS1 bioleaching groups under different pulp densities (1%, 5%, and 10% PD) at (**A**) 10 days, (**B**) 20 days, and (**C**) 30 days.

To examine functional-group changes in the leaching residues, FTIR characterization was performed on residues from each group at the early (10 d), middle (20 d), and final (30 d) stages of leaching (Figure 4). Across the samples, several common bands were observed. The sharp peak at 3677 cm^−1^ was assigned to O-H stretching vibrations of structural hydroxyl groups. The broad band at 3385–3428 cm^−1^ and the absorption at 1630–1655 cm^−1^ were associated with O-H stretching and H-O-H bending vibrations, respectively. The strong band at 1000–1020 cm^−1^ and the band at 625–669 cm^−1^ were assigned to SO_4_^2−^ vibrations in sulfate-bearing secondary phases, especially jarosite. The absorption at 461–467 cm^−1^ may be associated with metal–sulfur bond bending in residual minerals or FeO_6_ lattice vibrations of secondary iron minerals [13,14]. These assignments indicate the presence of hydroxyl- and sulfate-bearing species in the leaching residues.

Compared with the abiotic controls, the inoculated residues showed more evident jarosite-related infrared features during leaching. Jarosite-related absorptions appeared near 1082 cm^−1^ and in the range of 1199–1201 cm^−1^. At 10 d, the 1083 cm^−1^ peak was observed in the 5% and 10% pulp-density inoculated groups, whereas the abiotic groups showed only a single peak in this region. At 20 d, the doublet at 1082 and 1201 cm^−1^ became more distinct in the inoculated groups. An absorption band at 1427 cm^−1^ was also observed, which may be related to organic functional groups such as extracellular polymeric substances (EPS). By 30 d, the peaks at 1082 and 1199 cm^−1^ further intensified, and an absorption at 507 cm^−1^ appeared, which was assigned to FeO_6_ octahedral vibrations in jarosite. The O-H stretching band at 3392–3428 cm^−1^ also broadened and shifted toward lower wavenumbers. Together, these spectral changes are consistent with the accumulation of jarosite-related secondary phases in the inoculated residues.

The representative serpentine peak at 3677 cm^−1^ appeared consistently in residues from all groups, suggesting that serpentine-group gangue minerals persisted during leaching. In addition, an absorption band in the range of 795–800 cm^−1^ was observed only in the 1% PD_bio group at 10 d. According to Wang et al., this band can be assigned to Fe-O-H stretching vibrations in goethite and may represent an early secondary iron-mineral signal [15]. In the present study, this weak band was detected only at the early stage in the 1% PD_bio residue, whereas it was not clearly observed in the other groups or at later stages.

#### 2.2.4. Raman Spectral Analysis of Leaching Residue

**Figure 5:**
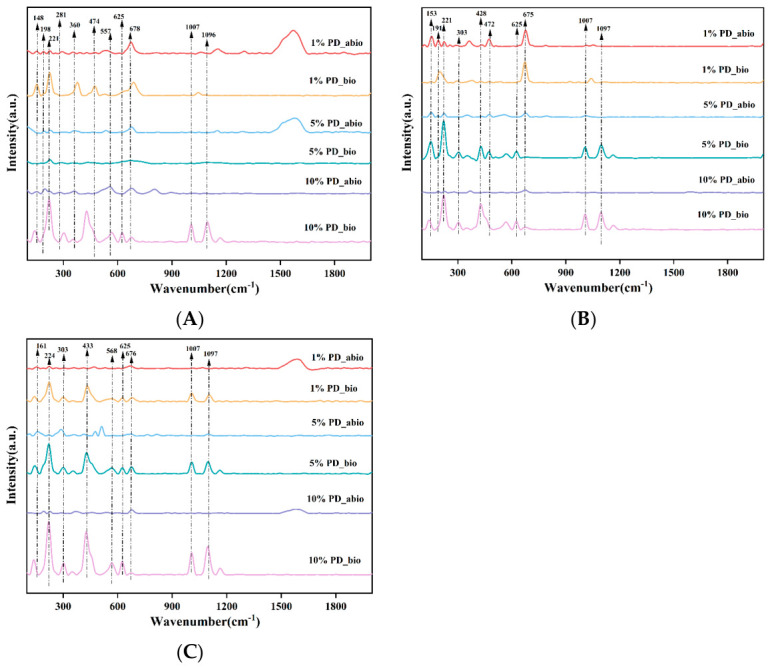
Raman spectra of the pentlandite leaching residues at (**A**) 10 days, (**B**) 20 days, and (**C**) 30 days at different pulp densities (1%, 5%, and 10% PD) for abiotic controls (sterile) and *A. ferriphilus* WGS1 bioleaching groups.

To further examine the composition and phase assemblage of pentlandite leaching products under different leaching conditions, Raman characterization was performed on residues from each group at the early (10 d), middle (20 d), and final (30 d) stages (Figure 5). In the spectra collected on day 10 (Figure 5A), low-frequency peaks at 148–153 cm^−1^ and 198 cm^−1^, together with a relatively strong band at 474 cm^−1^, were observed in the inoculated groups. These bands overlapped with the characteristic lattice vibrations of pentlandite at 145–155 cm^−1^, 200 cm^−1^, and 470–480 cm^−1^, suggesting that undissolved pentlandite residue was still present at this stage. Meanwhile, the band at 473 cm^−1^ can also be assigned to S-S symmetric stretching in cyclic octasulfur (alpha-S_8_), indicating the possible presence of elemental sulfur in the leaching residues.

As leaching proceeded to day 30 (Figure 5C), the low-frequency residual mineral peaks and the 474 cm^−1^ band weakened or disappeared, whereas the jarosite-related Fe-O lattice bands at 303 and 433 cm^−1^ and the gamma (OH) band at 568 cm^−1^ became more distinct. Overall, the spectra evolved toward more typical jarosite-related features. These changes suggest progressive alteration of pentlandite residues and accumulation of jarosite-type secondary phases during leaching. This interpretation is consistent with previous pentlandite bio-oxidation studies showing that mineral surface transformation can involve sulfur-rich species and jarosite-type Fe(III) precipitates during leaching [5].

In addition, compared with the abiotic controls, the inoculated groups showed stronger jarosite v1 (1007 cm^−1^) and v3 (1097 cm^−1^) bands at all time points, further supporting the formation of jarosite-related secondary phases in the inoculated residues.

#### 2.2.5. XPS Analysis of Leaching Residue

**Figure 6:**
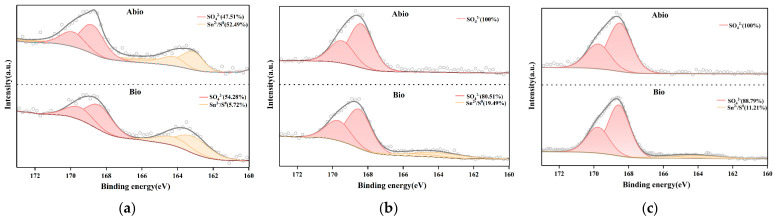

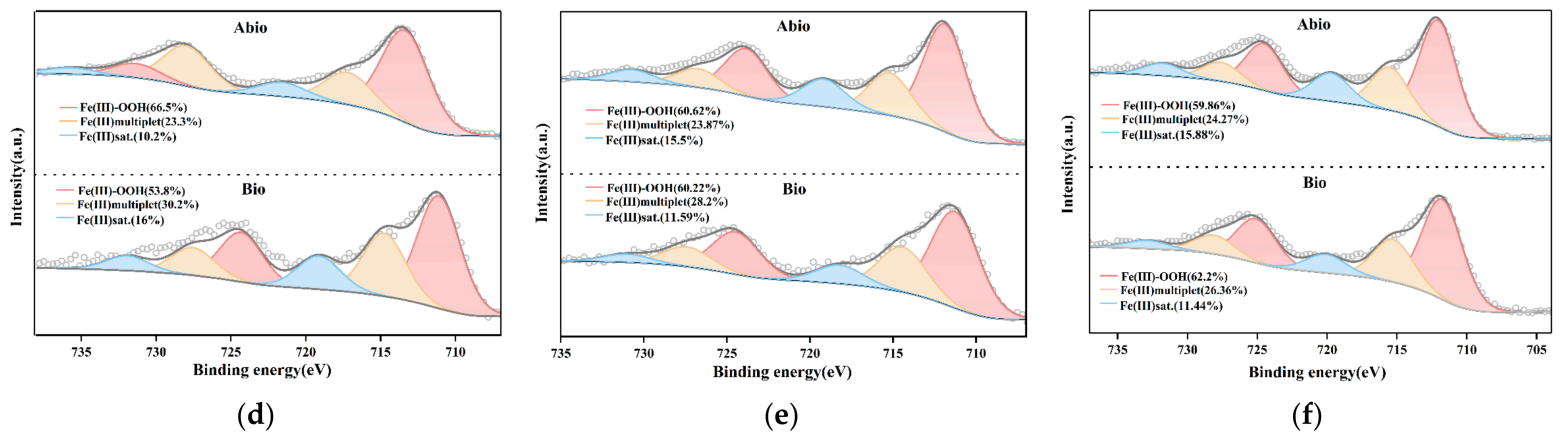
High-resolution X-ray photoelectron spectroscopy (XPS) spectra of the pentlandite leaching residues at different stages. (**a**–**c**) S2p core-level spectra on days 10, 20, and 30, respectively; (**d**–**f**) corresponding Fe2p core-level spectra. Abio and Bio denote the abiotic control and *A. ferriphilus* WGS1 bioleaching groups, respectively.

To further characterize surface chemical changes during pentlandite leaching, the 1% pulp-density group was selected for XPS analysis. This group showed the highest nickel leaching efficiency and relatively limited physical interference from high solid loading, allowing clearer comparison of Fe and S species on the residue surface. Figure 6a–c show the fitted S2p XPS spectra of the leaching residues. The spectra of all samples could be deconvoluted into two major sulfur species: sulfate (SO_4_^2−^) with binding energies in the range of 168–169 eV, and a combined peak of polysulfides and elemental sulfur (S_n_^2−^/S^0^) at 163–164 eV. These assignments are consistent with sulfur species previously reported during pentlandite bio-oxidation [5]. In the present study, SO_4_^2−^ was treated as an oxidized sulfur species, whereas the S_n_^2−^/S^0^ component was used to represent reduced or intermediate surface sulfur species.

At the early stage of leaching (Figure 6a), S_n_^2−^/S^0^ accounted for 52.49% in the abiotic control, while SO_4_^2−^ accounted for 47.51%. In the bioleaching group, the proportion of SO_4_^2−^ was 54.28%, whereas S_n_^2−^/S^0^ accounted for 45.72%. This result shows a slightly higher proportion of oxidized sulfur species in the inoculated residue at the early stage. At the middle stage (Figure 6b), the abiotic residue was fitted as SO_4_^2−^ only, whereas the bioleaching residue still contained 19.49% S_n_^2−^/S^0^. By the final stage of leaching (Figure 6c), the abiotic group still showed SO_4_^2−^ as the dominant fitted sulfur species, whereas the S_n_^2−^/S^0^ fraction in the bioleaching group further decreased to 11.21%. These temporal changes indicate different surface sulfur-speciation patterns between the abiotic and inoculated residues. In the inoculated residue, the decrease in the S_n_^2−^/S^0^ fraction from 45.72% to 11.21% is consistent with progressive transformation of reduced or intermediate sulfur species during leaching.

The Fe2p spectra are shown in Figure 6d–f. The binding energies of the main peaks in the six samples ranged from 711.11 to 711.75 eV. Together with the jarosite-related signals observed by Raman analysis, these Fe2p features are consistent with the presence of Fe(III)-bearing secondary phases, such as jarosite and/or FeOOH, on the residue surface. Similar secondary iron phases have also been reported during pentlandite bio-oxidation [5]. In addition, no obvious characteristic Fe^2+^ peaks were observed in the 706–710 eV region in any sample. This suggests that the detectable surface iron species were dominated by Fe(III)-bearing components under the tested conditions.

The fitted Fe2p components showed differences between the abiotic and inoculated residues during leaching. At the early leaching stage, the main-peak proportion in the Bio group was lower than that in the Abio group, whereas the proportion of the multiplet-splitting component was higher. The multiplet-splitting component was assigned to Fe(III)-related multiplet splitting based on reported Fe2p XPS fitting of iron compounds [16]. Therefore, the higher relative contribution of this component in the early Bio residue suggests a difference in the Fe(III)-bearing surface chemical environment compared with the corresponding abiotic residue. As leaching proceeded, the proportion of the multiplet-splitting component in the Bio group decreased, while the main-peak proportion increased. This trend suggests a temporal change in the Fe(III)-bearing surface chemical environment, consistent with the development of secondary iron phases during leaching. By contrast, the relative proportions of the fitted components in the Abio group remained comparatively stable across the three stages, suggesting less pronounced temporal variation in the surface Fe chemical environment of the abiotic residue.

### 2.3. Electrochemical Analysis

*A. ferriphilus* WGS1 maintained relatively high pentlandite leaching efficiency under high pulp-density conditions, indicating good tolerance and sustained bioleaching capacity. However, in practical industrial processes such as bioheap leaching and stirred-tank reactors, Ni^2+^ in solution often gradually accumulates to high levels, reaching tens of grams per liter at later stages of leaching. This accumulation can further affect oxidative dissolution and electron-transfer processes at the mineral interface. To more realistically simulate leaching behavior under industrial high-nickel conditions, this section analyzed the interfacial reaction characteristics of pentlandite in the presence of WGS1 under 40 g/L Ni^2+^ using electrochemical methods, including cyclic voltammetry, electrochemical impedance spectroscopy, and polarization curves.

(1) Cyclic voltammetry (CV)

**Figure 7:**
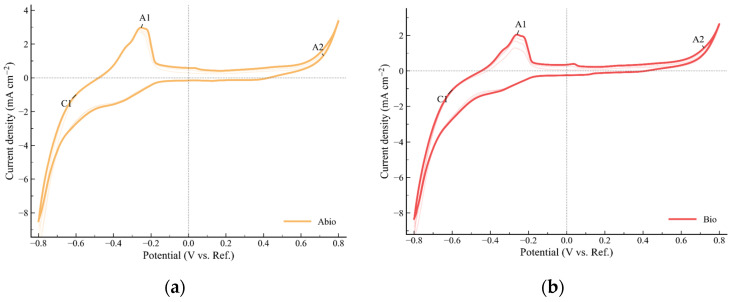

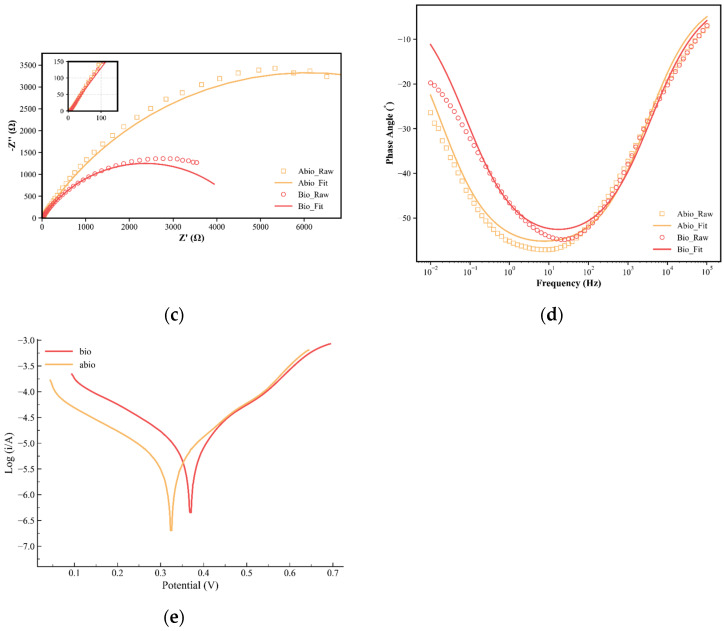
Electrochemical analysis of pentlandite during the bioleaching process by *A. ferriphilus* WGS1 under high-nickel conditions. Cyclic voltammetry (CV) curves of (**a**) the abiotic control and (**b**) the bioleaching group; (**c**) Nyquist plots and (**d**) Bode plots from electrochemical impedance spectroscopy (EIS); (**e**) Tafel polarization curves. Abio and Bio denote the abiotic control and the *A. ferriphilus* WGS1 bioleaching groups, respectively.

In the 40 g/L Ni^2+^ system, both the Bio and Abio pentlandite electrodes exhibited cyclic voltammetric responses, with a low-potential anodic peak A1, a high-potential anodic oxidation wave A2, and a cathodic peak C1 during the reverse scan (Figure 7a,b). A1, located at approximately −0.26 V, may correspond mainly to initial anodic dissolution and demetallization of the pentlandite surface, involving the preferential migration of Fe and Ni from the crystal lattice and the formation of sulfur-rich surface species. A2, located at approximately 0.72 V, may be associated with further oxidation of released Fe^2+^ and surface sulfur species, whereas C1, located at approximately −0.60 V, may reflect the reduction of partially oxidized surface species [17].

Compared with the Abio group, the open-circuit potential of the Bio group increased from 0.331 to 0.401 V, corresponding to a positive shift of approximately 70 mV. This positive shift indicates a higher interfacial potential in the WGS1-containing system. However, the A1 peak current decreased from 2.97 to 2.04 mA cm^−2^, and the CV loop area decreased from 1.866 to 1.422 mA V cm^−2^, suggesting that active dissolution-related charge transfer was partly restricted under the tested high-Ni^2+^ condition. Previous studies have shown that surface sulfur products and selective Fe/Ni migration during pentlandite oxidation can influence its electrochemical behavior [18]. Together with the residue characterization results, the CV features are consistent with changes in surface oxidation state and secondary-product accumulation in the Bio system.

(2) Electrochemical impedance spectroscopy (EIS)

The Nyquist plots (Figure 7c) showed that the impedance arc radius of the Bio group was smaller than that of the Abio group, indicating lower interfacial charge-transfer resistance in the WGS1-containing system. The equivalent-circuit fitting results (Table 1) showed that the solution resistance, Rs, was nearly identical between the two groups, with values of 5.88 Ohm for Bio and 5.869 Ohm for Abio, indicating only minor differences in bulk solution conductivity. In contrast, the Rct of the Bio group was 4720 Ohm, whereas that of the Abio group was 12,180 Ohm, representing an approximately 61.2% decrease. This result suggests that the presence of WGS1 was associated with lower charge-transfer resistance at the pentlandite interface under high-Ni^2+^ conditions.

Meanwhile, the Bode phase-angle plots (Figure 7d) showed one dominant phase-angle minimum for both samples, without multiple separated peaks. This suggests that, within the tested frequency range, the interfacial response could be fitted using the Rs(QRct) single-time-constant model. The broad phase-angle valleys and the minimum phase angle of approximately −55° indicated non-ideal capacitive behavior and surface heterogeneity. This was also supported by the fitted n values of 0.62–0.64, suggesting that both Bio and Abio interfaces were affected by surface roughness and/or secondary surface products. He et al. reported that jarosite and intermediate sulfur species can contribute to surface passivation during chalcopyrite bioleaching [19]. Bevilaqua et al. and Arena et al. also showed that bacterial participation can alter the impedance response at sulfide mineral electrode/solution interfaces [20,21]. In the present study, the lower Rct of the Bio group indicates more favorable charge-transfer behavior than in the Abio group, although surface heterogeneity was still evident.

(4) Tafel polarization curves

As shown in Figure 7e, the Tafel polarization curves of both the Bio and Abio groups exhibited cathodic and anodic regions, and the Bio curve shifted toward more positive potentials compared with the Abio curve. The fitting results (Table 2) showed that the corrosion potential, Ecorr, of the Bio group was 394.26 mV (vs. SSCE), representing a positive shift of approximately 60.30 mV relative to the Abio group (333.96 mV). The corrosion current density, jcorr, increased from 4.247 to 7.676 uA cm^−2^, corresponding to an increase of approximately 80.7%, while the polarization resistance, Rp, decreased from 8635.05 to 4751.95 Ohm cm^2^, a reduction of approximately 45.0%.

These electrochemical parameters indicate that the WGS1-containing system had a higher corrosion tendency and lower polarization resistance than the abiotic system under the tested high-Ni^2+^ condition. Meanwhile, the anodic Tafel slopes of the Bio and Abio groups were 0.1338 and 0.1366 V dec^−1^, respectively, and the cathodic Tafel slopes were 0.2257 and 0.2213 V dec^−1^, respectively. The small differences between the two groups suggest that the overall electrode reaction characteristics were similar, whereas the Bio system showed enhanced interfacial electrochemical activity. This result is consistent with the lower Rct observed for the Bio group in the EIS analysis and with the positive shift in open-circuit potential observed in the CV curves. Overall, the electrochemical results show that WGS1 was associated with lower charge-transfer resistance, higher corrosion current density, and a more positive interfacial potential, while surface-product effects under high-Ni^2+^ conditions may still have limited active dissolution sites.

### 2.4. Transcriptomic Responses Associated with High-Pulp-Density Bioleaching

**Figure 8:**
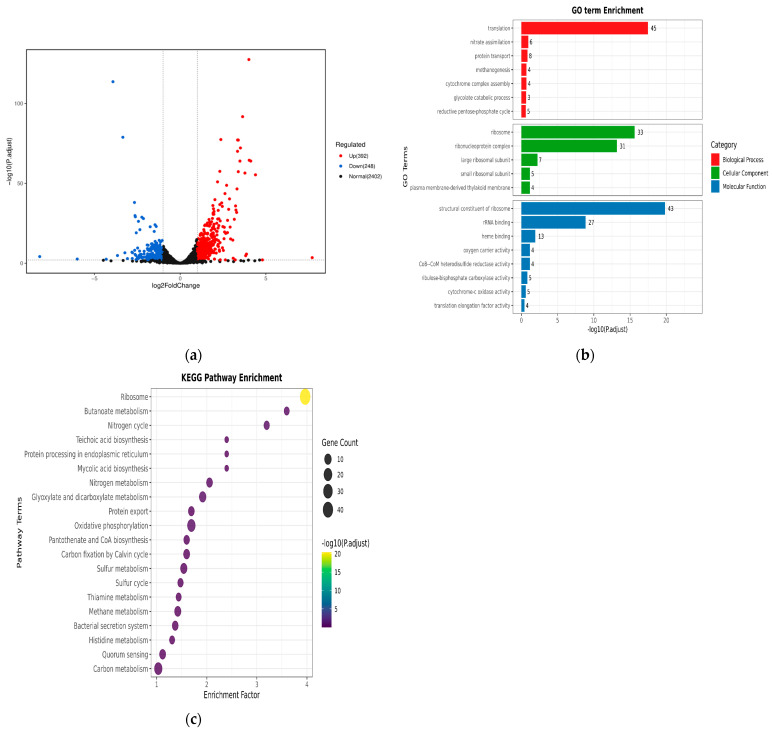
Transcriptomic analysis of *A. ferriphilus* WGS1 in response to high-pulp-density stress during pentlandite bioleaching. (**a**) Volcano plot of DEGs comparing the high-pulp-density group (experimental) to the low-pulp-density group (control). Red and blue dots indicate significantly up-regulated and down-regulated genes, respectively. (**b**) GO functional enrichment analysis and (**c**) KEGG pathway enrichment analysis of the DEGs. In (**c**), the size of each dot represents the gene count, and the color represents the −log_10_(P.adjust) value; dot sizes are scaled continuously and may not correspond exactly to the discrete values shown in the legend.

Bacterial cells were collected from the 1% and 10% (*w*/*v*) pulp-density bioleaching groups on day 30 and were designated the low-pulp-density (LPD) and high-pulp-density (HPD) groups, respectively. A total of 43.54 Gb of raw sequencing data was obtained, with Q30 values above 95% for all samples. Using an adjusted *p* value of ≤0.01 and an absolute log_2_fold change (|log_2_FC|) of ≥1 as the screening criteria, 640 differentially expressed genes were identified, including 392 upregulated and 248 downregulated genes. The volcano plot (Figure 8a) illustrates the overall transcriptional divergence between the HPD and LPD groups, providing a basis for subsequent analysis of candidate functional modules associated with cellular adaptation to the physicochemical environment of the HPD system.

To further clarify the biological significance of these differentially expressed genes, GO functional classification and KEGG pathway enrichment analyses were performed. GO enrichment showed that the DEGs were mainly associated with translation- and ribosome-related functions, including structural constituent of ribosome, translation, ribonucleoprotein complex, and rRNA-binding terms (Figure 8b). Because ribosome-associated processes are closely linked to bacterial stress responses [22,23], these enrichments indicate a major transcriptional adjustment of protein-synthesis-related functions under HPD conditions. KEGG enrichment further highlighted ribosome, oxidative phosphorylation, sulfur metabolism, carbon fixation, nitrogen metabolism/cycling, and quorum sensing (Figure 8c), consistent with broad metabolic and energy-related responses reported in metal-stressed or high-pulp-density bioleaching microorganisms [24,25,26].

#### 2.4.1. Candidate Responses Associated with Ni Homeostasis

**Figure 9:**
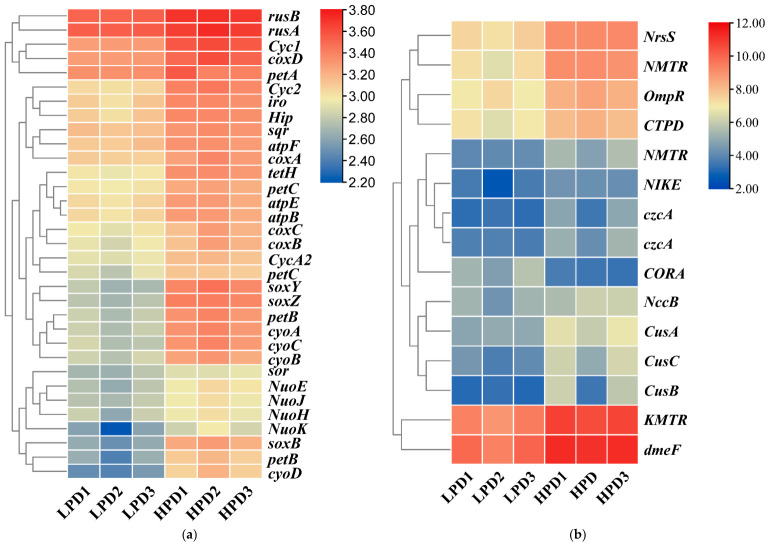
Heat map of differential expression of key functional genes in WGS1. ((**a**). Genes related to iron–sulfur oxidation and respiratory chain. (**b**). Genes related to nickel resistance.)

Under high-pulp-density conditions, the larger amount of pentlandite increased the mineral-associated buffering effect, metal-ion load, and potential interfacial constraints in the leaching system. In the present study, the HPD system showed higher dissolved Ni, Fe, and soluble sulfur levels than the LPD system, while WGS1 still maintained high Ni extraction efficiency. Therefore, transcriptomic comparison between the HPD and LPD groups was used to identify candidate cellular responses associated with Ni homeostasis, Fe/S oxidation, respiratory electron transfer, and energy conservation. These transcriptional changes were interpreted as molecular responses potentially related to adaptation under high-pulp-density and high-Ni conditions, rather than as direct evidence of pathway activity. To more clearly show the differences in transcriptional responses of key functional modules between the HPD and LPD conditions, representative differentially expressed genes associated with iron and sulfur oxidation, the respiratory chain, and nickel resistance were further screened and summarized (Table S1), and a clustering heatmap of these genes was generated (Figure 9).

As shown in Table S1 and Figure 9b, the genes annotated as *nrsS* (GE000260) and *ompR* (GE000085) showed log_2_FC values of 1.73 and 1.30, respectively, indicating higher transcript abundance in the HPD group than in the LPD group. NrsRS-type two-component systems have been reported to participate in Ni-responsive regulation in other bacteria [27]. The observed expression patterns therefore suggest the potential involvement of two-component regulatory responses in cellular adaptation to the combined physicochemical conditions of the HPD system. The specific inducing signals, regulatory targets, and functional consequences of these transcriptional changes in WGS1 remain to be determined.

Genome annotation identified a diverse repertoire of putative metal-transport systems in WGS1, including Nik/CorA-related uptake proteins, CDF-family transporters such as CTPD and DmeF, and Ncc/Cus-like efflux components. However, these genes exhibited heterogeneous transcriptional responses under the HPD condition, with differential expression confined to selected components rather than coordinated induction of the entire transport repertoire. Previous studies have shown that DmeF- and NcrABCY-like systems contribute to Ni/Co tolerance by regulating intracellular metal accumulation [28,29], while proteomic analyses of acidophilic bacteria have revealed broader reorganization of envelope and metal-efflux functions under metal stress [30]. Taken together, the genomic repertoire and selective expression patterns observed in WGS1 suggest that adaptation to the HPD condition may involve differential deployment of multiple metal-homeostasis modules rather than uniform activation of a single efflux pathway. Functional characterization of representative transporters and quantification of intracellular or cell-associated Ni will be required to determine their respective contributions.

#### 2.4.2. Ferrous Oxidation

In the iron-oxidation module, *iro* (GE001314), *rusA*, *rusB*, *cyc1*, and *cyc2* showed log_2_FC values of 1.81, 1.27, 1.52, 2.26, and 2.46, respectively, indicating higher transcript abundance in the HPD group than in the LPD group. In contrast, another *iro* copy (GE003013) and *cycA1* showed no reliable transcriptional signal in the present dataset. The responsive genes encode successive components of the canonical downhill electron-transfer chain associated with Fe^2+^ oxidation: Cyc2 functions as an outer-membrane electron acceptor, rusticyanins encoded by *rusA* and *rusB* mediate periplasmic electron transfer, and Cyc1 connects this electron flow to terminal oxidase-related components [31,32,33,34]. Iro/HiPIP-like proteins have also been proposed to participate in periplasmic electron distribution [35], while increased rusticyanin expression has been associated with enhanced Fe^2+^-oxidizing capacity in *A. ferrooxidans* [36]. Collectively, the coordinated transcriptional response of *iro*, *rusA*, *rusB*, *cyc1*, and *cyc2* is consistent with increased cellular investment in Fe-oxidation-associated electron-transfer components under the HPD condition. Such transcriptional adjustment may contribute to maintaining an oxidizing leaching environment as mineral loading and dissolved-metal concentrations increase, consistent with the high ORP maintained in the inoculated systems.

#### 2.4.3. Sulfur Oxidation

In the sulfur-oxidation module, *tetH*, *soxY*, *soxZ*, and *soxB* showed log_2_FC values of 2.24, 4.12, 4.00, and 3.64, respectively, indicating higher transcript abundance in the HPD group than in the LPD group. The genes *sqr* and *cycA2* also showed positive log_2_FC values of 1.26 and 1.40, respectively. By contrast, *sor* showed a log_2_FC value of 0.97 and therefore did not meet the stated differential-expression threshold of |log_2_FC| ≥ 1. The genes *sbp*, *doxDA*, *P21*, *sdo*, *omp40*, *soxA*, and *cyc* showed no reliable transcriptional signal in the present dataset. The WGS1 genome contains genes annotated as components of the S4I pathway, a truncated Sox system, and the SQR-SDO/SOR branch. The selective expression profile observed here indicates that the strongest transcriptional responses occurred in tetrathionate- and Sox-associated components, together with selected genes involved in sulfide oxidation and respiratory electron transfer. Chen et al. reported that sulfur oxidation in *Acidithiobacillus* does not rely on a single pathway but is jointly mediated by the truncated Sox system, the S4I pathway, and intracellular elemental-sulfur-processing branches [37]. Wang et al. further demonstrated that the *doxDA–tetH*-associated module is regulated by two-component systems [38]. Collectively, the coordinated responses of *tetH*, *soxY*, *soxZ*, *soxB*, *sqr*, and *cycA2* are consistent with transcriptional reorganization of sulfur-intermediate-processing functions under the HPD condition. This response may contribute to continued sulfur-species transformation in the inoculated system, in agreement with the accumulation of dissolved S and the temporal changes in surface sulfur speciation detected by XPS. The relative contributions of the individual sulfur-oxidation branches remain to be resolved by targeted biochemical and metabolic analyses.

#### 2.4.4. Respiratory Electron-Transfer Chain

The transcriptional response of respiratory-chain genes was also pronounced. The aa_3_-type cytochrome c oxidase genes *coxD, coxC, coxA*, and *coxB* showed log_2_FC values of 1.93, 1.50, 1.49, and 1.74, respectively. Among the two sets of bc1 complex-related genes, both the pet module containing GE000488-000490 and the GE003113-003118 module containing *Hip-CycA2* were upregulated, with *petB* (GE000489) and *petB* (GE003115) showing log_2_FC values of 3.28 and 2.88, respectively. In addition, the bo3-type terminal oxidase genes *cyoA-D* showed log_2_FC values ranging from 2.71 to 3.35, while the Complex I-related genes *nuoE, nuoH, nuoJ*, and *nuoK*, together with the ATP synthase genes *atpF* and *atpE*, also showed coordinated upregulation. Bruscella et al. showed that different pet modules in *A. ferrooxidans* may preferentially participate in either reverse electron transfer coupled to iron oxidation or forward electron transfer associated with sulfur oxidation [39]. The modeling analysis by Campodonico et al. further indicated that bc1 complexes, terminal oxidases, Complex I, and ATP synthase together form the core framework for electron distribution and energy conservation in acidithiobacilli [40]. Collectively, the coordinated increases in transcript abundance across the *cox*, *pet*, *cyo*, *nuo*, and *atp* modules indicate broad transcriptional adjustment of respiratory electron-transfer and energy-conservation components under the HPD condition. This expression pattern is consistent with previous observations that metal-sulfide pulp density can affect the transcription of electron-transfer-related genes in *Acidithiobacillus* [26]. Such transcriptional adjustment may facilitate electron-flow redistribution and support proton-motive-force-dependent cellular processes as solid loading and dissolved-metal concentrations increase, thereby providing a plausible molecular context for the sustained bioleaching performance of WGS1 under the HPD condition.

### 2.5. Integrated Analysis of Ni Extraction Performance and Bioleaching Mechanism

As summarized in Table 3, previously reported pentlandite and nickel-sulfide bioleaching systems have been evaluated under different conditions, including chemical enhancement, mixed microbial cultures, continuous reactors, and elevated temperatures [5,7,8,41,42,43]. Because these studies differ in mineral composition, microbial systems, pulp density, temperature, and leaching duration, their extraction efficiencies should be compared with caution. Under the conditions tested in the present study, WGS1 achieved 95.7% Ni extraction at 10% pulp density and 35 °C after 30 days. This result indicates that WGS1 maintained high pentlandite leaching performance under the tested mesophilic and relatively high-pulp-density conditions. Further studies in stirred-tank reactors, continuous systems, and complex mineral feedstocks are required to evaluate its scale-up potential and long-term performance.

To interpret this leaching phenotype, the integrated datasets provide complementary information at different levels. Ni extraction, solution chemistry, mineralogical and surface characterization, and electrochemical parameters describe the observed leaching performance and associated interfacial changes. Genome annotation and differential transcript abundance provide molecular context for identifying candidate cellular responses. Integration of these datasets supports a literature-informed working model involving Ni homeostasis, Fe/S oxidation, and respiratory electron transfer under the HPD condition.

**Figure 10:**
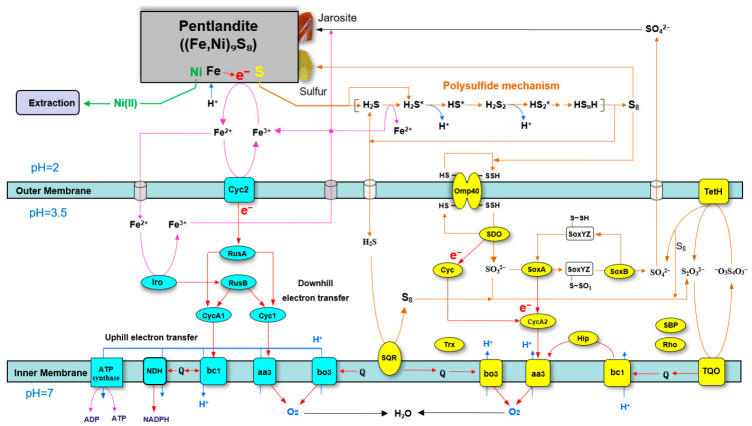
The putative mechanism model of pentlandite bioleaching by *A. ferriphilus* WGS1. In the figure, pink arrows indicate the downhill electron transfer pathway via iron oxidation; orange arrows represent the sulfur/polysulfide oxidation pathway; red arrows with “e^−^” indicate electron flow direction; the green arrow indicates the release and extraction of Ni^2+^. Cyan-colored boxes/ovals represent cytochrome proteins, while yellow-colored boxes/ovals represent oxidoreductase enzymes and respiratory complexes. The degradation of pentlandite results from oxidative attack of ferric ions (Fe^3+^) or acidic dissolution of hydrogen ions (H^+^) on the mineral surface. In this interfacial redox reaction, ferric ions are reduced into ferrous ions (Fe^2+^) by the electrons from pentlandite, while the elements of Ni and S of pentlandite are gradually released into the bioleaching solution from the surface. The released soluble nickel ions (Ni^2+^) are extracted and thus achieve the industrial purpose of nickel metallurgy. The released sulfur is caused by a polysulfide mechanism, in which the metal sulfide is degraded via polysulfides as key intermediates. Firstly, the released sulfur may generate hydrogen sulfide (H_2_S), which is then subjected to one-electron oxidation to produce the cation radical H_2_S* by the ferric ion. The cation radical may also be directly formed on the surface of pentlandite by the attack of the ferric ion. Secondly, by dissociation of the strong acid H_2_S*, the radical HS* occurs. Two of these radicals may react to form a disulfide (H_2_S_2_), which further releases H^+^ to form the radical disulfide ion (HS_2_*). Tetrasulfide can occur by dimerization of two HS_2_* or trisulfide by reaction of HS* with HS_2_* radicals. Chain elongation of the polysulfides may proceed by analogous reactions. In acidic solutions, polysulfides decompose to release H_2_S, liberating rings of elemental sulfur, mainly S_8_. The role of *A. ferriphilus* in pentlandite bioleaching is to oxidize these ferrous ions and various reduced inorganic sulfur compounds (RISCs), mainly hydrogen sulfide (H_2_S) and elemental sulfur (S_8_). The oxidation of Fe^2+^ leads to the regeneration or recycling of the oxidant of Fe^3+^. The oxidation of RISCs results in the transformation of insoluble or inhibiting intermediates into the final highly soluble product of sulfate (SO_4_^2−^), thereby promoting the progress of pentlandite bioleaching. The iron oxidation system of *A. ferriphilus* consists of downhill and uphill electron transfer pathways. The downhill pathway is from the oxidation of extracellular Fe^2+^ by Cyc2 or periplasmic Fe^2+^ by Iro and then successively transfers electrons to rusticyanins (RusA and RusB), Cyc1, the aa_3_ complex, and O_2_. The uphill pathway pushed by the transmembrane gradient proton is bifurcated from rusticyanins (RusA and RusB) and then successively transfers electrons to CycA1, reverse bc_1_ complex, quinone pool, reverse NDH, and NADPH. The sulfur oxidation system of *A. ferriphilus* mainly contains Omp40, SDO, SQR, SoxABYZ, TQO, TerH, various cytochrome c, etc. The extracellular H_2_S enters the periplasmic space via the outer membrane pore channel. SQR catalyzes H_2_S to produce sulfane sulfur (S_n_) and puts an electron into the quinone pool. The extracellular elemental sulfur (S_8_) is activated by thiol groups of a special outer-membrane protein (Omp40) and transported into the periplasmic space as persulfide sulfur (R-SSH), and then oxidized to produce sulfite (SO_3_^2−^) and bring an electron to the aa_3_ complex via Cyc, CycA2 by the sulfur diooxygenase (SDO). SoxYZ binds SO_3_^2−^ with the help of SoxA to produce the SoxYZ-thiosulfate adduct and bring an electron to the aa_3_ complex via CycA2. SoxB splits this adduct to generate the final product of sulfate. Sulfur and sulfite can non-enzymatically and reversibly be placed into thiosulfate. TQR catalyzes thiosulfate to produce tetrathionate (O_3_^2−^SSSSO_3_^2−^) and puts electrons into the quinone pool. TetH hydrolyzes tetrathionate to produce thiosulfate, sulfur, and sulfate. The forward bc_1_ complex extracts electrons from the quinone pool and then transfers them to the aa_3_ complex via Hip to reduce oxygen into water. The bo_3_ complex directly extracts electrons from the quinone pool to reduce oxygen. Under the actions of various respiratory components, the transmembrane proton gradient is formed and thus drives ATP synthetase and reverse NADH dehydrogenase (NDH) to produce ATP and NADPH, which provide energy and electrons for the fixation of carbon dioxide (CO_2_) and nitrogen (N_2_) for the growth and reproduction of this chemoautotroph. During the bioleaching process, as the concentrations of Fe^3+^ and SO_4_^2−^ in the system increase, jarosite may precipitate; the jarosite, together with the generated elemental sulfur (S^0^), deposits on the surface of the mineral, forming a dense passivation layer. This layer significantly inhibits the attachment and oxidation of microorganisms to the mineral matrix, thereby hindering the continuous progress of the bioleaching reaction.

The high Ni extraction observed in the WGS1-containing system under 10% pulp density may reflect more than nickel tolerance alone. Transcriptomic patterns showed changes in genes annotated for nickel homeostasis, iron/sulfur oxidation, and respiratory-chain electron transfer, but these data should be interpreted as molecular context rather than direct evidence of pathway activity. Based on genome annotation, transcriptomic patterns, and the observed physicochemical and electrochemical changes, we proposed a tentative working model for WGS1-associated pentlandite bioleaching, as shown in Figure 10. Overall, these responses may be associated with nickel adaptation, Fe/S-related interfacial processes, and energy metabolism under the tested high-pulp-density conditions.

As pentlandite dissolution proceeded, Ni^2+^ progressively accumulated in the system, while secondary products such as jarosite, FeOOH, and sulfur species were gradually deposited on the mineral surface. The high solid loading may have further intensified mass-transfer limitations. These factors could affect microbial activity, interfacial electron transfer, and oxidative mineral dissolution. Previous studies have shown that excess Ni^2+^ can affect microbial metabolism by disrupting metal homeostasis, inhibiting metalloenzyme activity, and inducing oxidative stress [10]. Therefore, the high Ni leaching observed in the WGS1-containing system may be related to a coordinated physiological response involving nickel homeostasis, Fe/S-related redox processes, and respiratory energy metabolism, rather than to nickel tolerance alone. However, because intracellular Ni^2+^ accumulation, ROS status, transporter activity, and stress-related protein abundance were not directly quantified during pentlandite bioleaching, this interpretation should be regarded as a transcriptome-supported working model rather than direct physiological proof.

The solution chemistry and mineralogical results were consistent with greater pentlandite alteration in the WGS1-containing systems than in the sterile controls. After the initial buffering stage, the inoculated groups maintained lower pH and higher ORP than the sterile controls, whereas Abio-10% showed persistently higher pH and lower ORP. SEM-EDS, XRD, FTIR, and Raman analyses further showed more extensive surface alteration and secondary-phase formation in the WGS1-containing systems. However, because jarosite, FeOOH, and sulfur species were also detected, surface-product accumulation may still have affected the leaching interface rather than being fully overcome by WGS1-associated processes.

The XPS results revealed temporal changes in surface sulfur speciation during pentlandite leaching. In the inoculated residues, the relative contribution of the S_n_^2−^/S^0^ component decreased from 45.72% at the early stage to 11.21% at the late stage, accompanied by a predominance of the SO_4_^2−^ component. These changes are consistent with progressive transformation of reduced or intermediate surface sulfur species during bioleaching. However, because the fitted S_n_^2−^/S^0^ component represents a combined spectral contribution from polysulfide and elemental sulfur species, the individual sulfur intermediates involved in this transformation could not be distinguished by XPS.

These surface sulfur-speciation changes can be interpreted within the established polysulfide mechanism for acid-soluble metal sulfides [3,5,44]. In this reaction framework, Fe^3+^- and H^+^-associated attack on the pentlandite surface initiates metal release and the formation of reduced sulfur intermediates. Successive one-electron oxidation, deprotonation, and radical-coupling reactions may generate H_2_S*, HS*, H_2_S_2_, HS_2_*, and higher-chain polysulfide species, which are subsequently transformed into elemental sulfur, thiosulfate, tetrathionate, and sulfate. The temporal decrease in the combined S_n_^2−^/S^0^ contribution is compatible with continued transformation of these reduced or intermediate sulfur pools in the inoculated residues.

Accordingly, the polysulfide route was incorporated into Figure 10 as a literature-derived reaction framework linking pentlandite dissolution with microbial Fe/S oxidation. Genome annotation and transcriptomic patterns further identified candidate components associated with Fe^2+^ oxidation and sulfur processing, including Cyc2, rusticyanins, SQR, Sox proteins, and TetH/TQO. Integration of these components with the mineralogical, surface-chemical, and electrochemical observations provides a working model for sulfur transformation during WGS1-associated pentlandite bioleaching.

The electrochemical results further showed different interfacial electrochemical behavior between the WGS1-containing and abiotic systems under high-Ni^2+^ conditions. In the 40 g/L Ni^2+^ system, the WGS1-containing system showed positive shifts in open-circuit potential and corrosion potential, lower Rct, higher corrosion current density, and lower polarization resistance than the abiotic control. These results indicate lower apparent charge-transfer resistance and a greater oxidative tendency at the pentlandite interface. However, the decreased A1 peak current and CV loop area suggest that secondary products such as jarosite, FeOOH, and sulfur species may still have masked part of the active surface. Thus, WGS1 was associated with improved, but not passivation-free, interfacial oxidation under the tested high-Ni^2+^ condition.

The transcriptomic results provide a molecular context for interpreting the observed phenotypes rather than direct evidence of pathway activity. Under high-pulp-density and high-Ni conditions, coordinated expression changes were observed in genes associated with Ni^2+^ sensing and transport, Fe^2+^ oxidation, sulfur-intermediate processing, and respiratory-chain energy conservation. Changes in genes related to *NrsS/OmpR, CTPD, dmeF*, and *Ncc/Cus* suggest the potential involvement of multiple Ni-homeostasis modules [27,28,29]. Similarly, the expression patterns of *iro*, *rusA*/*rusB*, *Cyc1*/*Cyc2*, *tetH*, *soxY*, *soxZ*, *soxB*, *sqr*, *sor*, *cox*, *pet*, *cyo*, *nuo*, and *atp* are consistent with the possible involvement of Fe/S oxidation-related processes and respiratory-chain energy conservation [45,46]. However, transcript abundance alone does not demonstrate increased protein abundance, transport activity, or metabolic flux. Future work should combine intracellular or cell-associated Ni quantification, in situ ROS measurements during bioleaching, proteomic or enzymatic analyses, and functional validation of representative transporters to further test this working model.

Overall, WGS1-mediated pentlandite bioleaching can be interpreted as a coupled process involving Ni homeostasis, Fe/S oxidation, and interfacial electron transfer. Mineralogical and surface-chemical analyses showed progressive pentlandite alteration, secondary Fe/S phase formation, and sulfur-speciation changes, while electrochemical results showed reduced charge-transfer resistance and enhanced oxidative tendency under high-Ni^2+^ conditions. Transcriptomic results further suggested coordinated responses involving metal homeostasis, Fe^2+^ oxidation, sulfur-intermediate processing, and respiratory-chain energy conservation. Together, these observations provide a working explanation for the high Ni extraction achieved by WGS1 at 10% (*w*/*v*) pulp density, while supporting further evaluation in more complex nickel-sulfide feedstocks.

The present study was conducted in shake flasks over a 30-day period and should therefore be regarded as a laboratory-scale investigation. In addition, although pentlandite was the dominant sulfide phase in the mineral sample, with a small amount of lizardite also present, this sample does not fully represent the complexity of industrial nickel-sulfide concentrates. In mixed-mineral systems, additional sulfide minerals, such as pyrrhotite and chalcopyrite, and variable gangue minerals may affect galvanic interactions, impurity-metal release, acid consumption, slurry rheology, secondary-mineral precipitation, and microbial activity. Consequently, the high Ni extraction obtained in the present study should not be directly extrapolated to industrial feedstocks or large-scale operation.

Future work should evaluate representative mixed-mineral concentrates in bench-scale stirred-tank or continuous bioleaching systems. Key points should include Ni extraction and selectivity, pH and ORP control, Fe^2+^/Fe^3+^ speciation, sulfur-intermediate transformation, oxygen transfer, slurry behavior, secondary-mineral deposition, and the stability of WGS1 activity over successive cycles. In addition, targeted proteomic, enzymatic, or metabolic analyses are needed to test the Fe/S oxidation routes proposed in the working model.

## 3. Materials and Methods

### 3.1. Mineral Samples and Characterization

**Figure 11:**
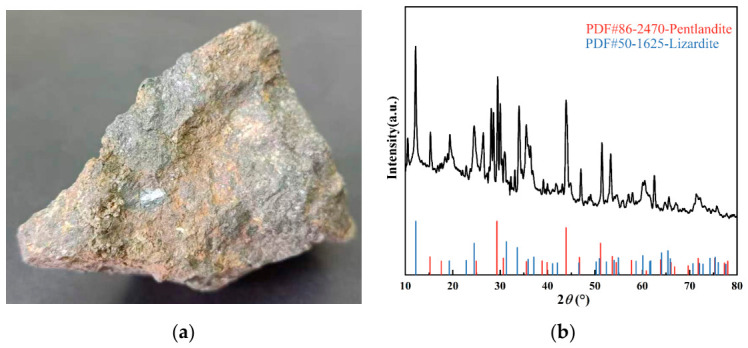
The morphology and mineral phase analysis of ore samples. (**a**) The morphology of pentlandite ore; (**b**) the XRD pattern of pentlandite ore.

The pentlandite used in this study was provided by the School of Minerals Processing and Bioengineering, Central South University, Changsha, China. The mineral particles were crushed and then ground through 200–400 mesh sieves, ensuring that most powder particles were within the size range of 38–75 µm. After dissolution in aqua regia, the elemental contents of Fe, S, and Ni in the pentlandite sample were determined by inductively coupled plasma optical emission spectrometry (ICP-OES, iCAP 7400, Thermo Fisher Scientific, Waltham, MA, USA) to be 21.77%, 9.85%, and 6.65%, respectively. The phase composition of the sample was analyzed by X-ray diffraction (XRD; Rigaku MiniFlex 600, Rigaku Corporation, Tokyo, Japan) using Cu Kα radiation over a scanning range of 10–80° at a scanning rate of 5°/min. The results (Figure 11b) showed that the pentlandite sample contained a small amount of lizardite (Mg_3_Si_2_O_5_(OH)_4_), with pentlandite ((Fe,Ni)_9_S_8_) as the dominant sulfide phase, meeting the requirements of this study.

### 3.2. Bacterial Strain and Culture Condition

*Acidithiobacillus ferriphilus* WGS1 was used as the bioleaching strain in this study. This strain was isolated in our laboratory from acid mine drainage collected at a nickel- and iron-sulfide-rich mineral waste site in the Wugaishan mine, Chenzhou, Hunan Province, China. It exhibits high nickel tolerance and is preserved in the Key Laboratory of Biometallurgy of the Ministry of Education, China. Whole-genome sequencing of this strain was also performed in our laboratory, and the sequencing data have been deposited in the NCBI database under accession number GCA_055768765.1, with BioSample ID SAMN56136976. The 9K medium was used as the basal medium for cultivation. The 9K medium contained, per liter of water, 3.0 g (NH_4_)_2_SO_4_, 0.1 g KCl, 0.5 g K_2_HPO_4_, 0.5 g MgSO_4_·7H_2_O, and 0.01 g Ca(NO_3_)_2_. Unless otherwise stated, this basal medium was an iron-free 9K salt medium, and no soluble ferrous or ferric salts were added to the bioleaching medium. Thus, dissolved Fe detected during the bioleaching experiments mainly originated from pentlandite dissolution rather than from externally added iron. The initial pH of the medium was adjusted to 2.0 with 1 M H_2_SO_4_. All chemical reagents used in the medium were purchased from Sinopharm Chemical Reagent Co., Ltd. (Beijing, China).

For routine cultivation, WGS1 was maintained aerobically in 9K medium supplemented with 44.7 g/L FeSO_4_·7H_2_O as the energy source. The initial pH was adjusted to 2.0 with 1 M H_2_SO_4_, and the cultures were incubated at 35 °C with shaking at 180 rpm. The strain was transferred to fresh medium every 3–5 days using a 10% (*v*/*v*) inoculum. For long-term preservation, the strain was stored at −80 °C in 20% (*v*/*v*) glycerol.

### 3.3. Bioleaching Experiments

Before the bioleaching experiments, the strain was repeatedly acclimated in basal medium containing 44.7 g/L ferrous sulfate heptahydrate (FeSO_4_·7H_2_O) by gradually increasing the concentration of nickel sulfate (NiSO_4_), ultimately obtaining a subculture resistant to 40 g/L Ni^2+^. This subculture was then adapted to pentlandite by adding 10 g/L pentlandite sample to the basal medium and culturing it continuously for several generations. Pentlandite bioleaching by *Acidithiobacillus ferriphilus* WGS1 was conducted in 250 mL Erlenmeyer flasks containing 100 mL basal medium supplemented with pentlandite. Pulp density was defined as the mass of pentlandite sample added per unit volume of the leaching medium (% *w*/*v*). Accordingly, pulp densities of 1%, 5%, and 10% (*w*/*v*) corresponded to 10, 50, and 100 g/L, respectively, equivalent to the addition of 1, 5, and 10 g of pentlandite to 100 mL of basal medium. These pulp densities were selected to represent a low-solid baseline, an intermediate loading, and a relatively high-solid challenge condition, thereby enabling evaluation of the bioleaching performance of WGS1 as mineral buffering, Ni^2+^ accumulation, and mass-transfer constraints increased with solid loading. The selected range also covers pulp densities used in previous nickel-sulfide bioleaching studies [7,8]. The flasks were incubated in an aerobic shaking incubator (ZQZY-108B, Shanghai Zhicheng Analysis Instrument Manufacturing Co., Ltd., Shanghai, China) at 35 °C and 180 rpm. The initial cell density for each experiment was 1 × 10^8^ cells/mL. All experiments were performed in triplicate under identical conditions. Abiotic control experiments were conducted under the same conditions.

The bioleaching experiments were conducted for 30 days. Solution samples were collected at 3-day intervals on days 0, 3, 6, 9, 12, 15, 18, 21, 24, 27, and 30 to monitor pH, oxidation–reduction potential (ORP), cell concentration, soluble Ni, total Fe, and soluble S. Leaching residues were collected on days 10, 20, and 30 for mineralogical and surface characterization. The pH and ORP values were measured with a pH meter and a platinum electrode with a Ag/AgCl reference electrode (PHS-3E, INASE Scientific Instrument Co., Ltd., Shanghai, China). Cell density was directly counted using a light microscope (Olympus, Center Valley, PA, USA) with a blood corpuscle counter (XB-K-25). The concentrations of dissolved S and Ni in the leaching solutions were quantified by inductively coupled plasma optical emission spectrometry (ICP-OES, IRIS Intrepid II XSP, Thermo Fisher Scientific, Waltham, MA, USA). The concentrations of total Fe ([Fe^T^]aq) were determined using 5-sulfosalicylic acid spectrophotometry [47].

### 3.4. Leaching Residue Analysis

The surface morphology and elemental composition of the pentlandite leaching residue samples collected at different leaching stages were characterized by scanning electron microscopy (SEM) coupled with energy-dispersive X-ray spectroscopy (EDS) (Nova™ NanoSEM 230, FEI, Hillsboro, OR, USA). Fourier transform infrared spectroscopy (FTIR) analysis was performed with a Fourier transform spectrometer (Nexus 670, Nicolet, Madison, WI, USA). X-ray diffraction (XRD) analysis was conducted with CuKα radiation (40 kV/250 mA) in a RINT2000 vertical goniometer. The scanning range was from 10° to 80° 2θ with a step of 0.02° and a dwell time of 4 s. Mineral composition analysis was performed using a Raman spectrometer (Renishaw InVia Qontor, Renishaw plc, Wotton-under-Edge, UK). The mineral samples of the substrate residue were placed on the stage of an Olympus BHSM microscope (Olympus Corporation, Tokyo, Japan) and oriented. The Raman spectra were excited by a He–Ne laser (633 nm) with a resolution of 2 cm^−1^ and in the range of 100 to 4000 cm^−1^. The Fe and S speciation transformations on the mineral surfaces were analyzed by X-ray photoelectron spectroscopy (XPS) with an excitation source of Al Kα X-ray (1486.6 eV) at 12 kV and 6 mA, and the data was processed using Avantage software version 6.6 (Thermo Scientific, Waltham, MA, USA). After aligning the C 1s peak with 284.8 eV, all spectra were fitted and calculated based on existing research results.

### 3.5. Electrochemical Investigations

Electrochemical measurements were performed in 9K medium (pH 2.0) using an INTERFACE 1010E electrochemical workstation (Gamry; Reference 600 & Interface 1010E with RED710) in a conventional three-electrode system. A saturated calomel electrode (SCE) was used as the reference electrode, a platinum electrode as the counter electrode, and the mineral electrode as the working electrode. The mineral electrode was prepared by mixing 1.05 g of mineral sample, 0.30 g of graphite, and 0.15 g of paraffin, followed by pressing at 120 kg for 10 min. After grinding and polishing, the working electrode was immersed in 9K medium, and the open-circuit potential (OCV) was first recorded. Electrochemical impedance spectroscopy (EIS) was conducted after the OCV became stable. During the measurements, the bacterial concentration in the inoculated 9K medium was approximately 1 × 10^8^ cells/mL, whereas no bacteria were added to the sterile control. Ni^2+^ was added to all electrochemical cells at a concentration of 40 g/L. EIS was performed over a frequency range of 0.05–10^5^ Hz with a sinusoidal perturbation amplitude of 10 mV. Tafel polarization curves were recorded over a potential range from −300 mV versus OCV to +300 mV versus OCV at a scan rate of 1 mV/s. Cyclic voltammetry was performed at a scan rate of 10 mV/s, scanning first from the OCP to 850 mV, then reverse-scanning to −750 mV, and finally returning to the OCP.

### 3.6. Genomic Bioinformatics and Transcriptomics Analysis

Bacterial cells from the low-pulp-density (1%) and high-pulp-density (10%) experimental groups were collected for microbial transcriptome sequencing. The harvested cells were washed with PBS buffer and pelleted by high-speed centrifugation, immediately frozen in liquid nitrogen, and stored at −80 °C. Total RNA was extracted using the TRIzol method, followed by sample quality control. After the samples passed quality assessment, ribosomal RNA was removed, cDNA libraries were constructed, and qualified libraries were sequenced. RNA quality control, library construction, and sequencing were performed by Tsingke Biotechnology Co., Ltd. (Beijing, China). Bioinformatic analysis was conducted using the previously obtained WGS1 whole-genome data as the reference genome. Gene expression was quantified using RSEM [48], and differentially expressed genes were identified using DESeq2 [49]. Sequenced genes were aligned against the GO and KEGG databases, and differentially expressed genes were subjected to functional classification, annotation, and enrichment analysis.

### 3.7. Statistical Analysis

All experiments were carried out at least in triplicate under the same conditions. The experimental data were analyzed by one-way analysis of variance in SPSS 20.0 software, and values of *p* < 0.05 were considered statistically significant. The results were statistically presented in terms of the mean value with the standard deviation (SD) as the error bar from repeat data by using Origin 2024 and Excel 2024 software.

## 4. Conclusions

This study showed that the Ni-tolerant acidophile *Acidithiobacillus ferriphilus* WGS1 achieved efficient pentlandite bioleaching at relatively high pulp densities under the tested conditions. After 30 d of leaching at 35 °C, Ni leaching efficiencies in the biotic systems reached 99.2%, 97.1%, and 95.7% at pulp densities of 1%, 5%, and 10%, respectively, whereas the abiotic controls showed only limited mineral dissolution. The WGS1-containing systems restored acidity after mineral buffering and maintained higher ORP than the sterile controls over an extended period, indicating solution conditions favorable for oxidative mineral dissolution under high solid loading and high Ni^2+^ stress.

Integrated mineralogical, surface-chemical, electrochemical, and transcriptomic analyses provided complementary perspectives on WGS1-associated pentlandite bioleaching. Mineral residues showed pentlandite alteration, Ni depletion, secondary Fe/S phase formation, and temporal changes in surface sulfur speciation. Under 40 g/L Ni^2+^, the WGS1-containing system exhibited lower charge-transfer and polarization resistances and a higher corrosion current density than the abiotic system, consistent with more favorable interfacial electrochemical behavior.

Transcriptomic comparison between the HPD and LPD groups identified coordinated changes in genes annotated for Ni homeostasis, Fe/S oxidation, respiratory electron transfer, and energy conservation. These expression patterns provide molecular context for the bioleaching phenotype and support a literature-informed working model involving metal homeostasis and Fe/S-related electron-transfer processes under the HPD condition. Further functional studies integrating intracellular or cell-associated Ni quantification, ROS measurements, transporter characterization, and protein- or metabolite-level analyses will help resolve the contributions of the candidate pathways. The present findings establish the laboratory-scale bioleaching performance of WGS1 and support its further evaluation using mixed-mineral feedstocks and controlled reactor systems.

## Figures and Tables

**Table 1:** Equivalent circuit fitting table.

System	Equivalent Circuit	Rs (Ω)	Rct (Ω)	*n*
Abio	Rs(QRct)	5.869	12,180	0.6363
Bio	Rs(QRct)	5.88	4720	0.62

**Table 2:** Polarization curve parameters.

System	Ecorr (mV vs. SSCE)	Jcorr (uA cm^−2^)	Rp (ohm cm^2^)	Anodic Slope (V/dec)	Cathodic Slope (V/dec)
Bio	394.26	7.676	4751.95	0.1338	0.2257
Abio	333.96	4.247	8635.05	0.1366	0.2213

**Table 3:** Comparison of the ore-leaching effects and nickel resistance capabilities of different ore-leaching bacteria.

Bacterial Strains	Minerals	Condition	Pulp Density (%)	Day	Leaching Rate (%)	Ni^2+^ Concentration (mM)	References
*A. ferrooxidans*	Pentlandite (Ni 0.5%, Fe 2.51%)	Cultivation with bubble stirring at 30~70 °C	10	15	83.8	7.14	[43]
Mixed bacteria	Pentlandite (Ni 27.43%, Fe 35.13%)	Stir and culture at 33 °C	2	16	40	37.39	[5]
Moderately thermophilic mixed microbial community	Pentlandite (Ni 9.0%, Fe 32.3%)	Stir and culture at 48–49 °C	9.8	9	66	99.19	[8]
Moderately thermophilic mixed microbial community	Nickel sulfide concentrate (Ni 1.9%)	Stir and culture at 68 °C	10	4	98	31.73	[42]
*L. f* + *A. f* + *A. t*	Nickel-iron concentrate (Ni 5.9%, Fe 28.1%)	shake flask at 34 °C and 200 rpm	5	30	52	50.26	[7]
*Alicyclobacillus* spp.	Violarite (Ni 7.2%, Fe 21.8%)	shake flask at 40 °C and 180 rpm	1	22	97	11.9	[7]
*T.ferrooxidans*	Pentlandite (Ni 32.72%, Fe 21.8%)	shake flask at 35 °C and 170 rpm	2	5	25	27.9	[41]
*A. ferriphilus WGS1*	Pentlandite (Ni 6.65%, Fe 21.77%)	shake flask at 35 °C and 180 rpm	10	30	95.7	110.47	This study

## Data Availability

The whole-genome sequence data of *Acidithiobacillus ferriphilus* WGS1 are available in the NCBI database under accession number GCA_055768765.1, with BioSample ID SAMN56136976. The raw RNA-seq data generated in this study have been deposited in the NCBI Sequence Read Archive (SRA) under BioProject accession number PRJNA1429581 and SRA study accession number SRP704731 (run accessions SRR38893967–SRR38893972). Functional annotation and enrichment analyses were performed using the Gene Ontology (GO) and Kyoto Encyclopedia of Genes and Genomes (KEGG) databases. Processed transcriptomic data supporting the conclusions of this article are included in the article and its Supplementary Materials.

## Supplementary Materials

The following supporting information can be downloaded at: https://www.mdpi.com/article/10.3390/ijms27135762/s1.
