# Supplementary material for: Integrative Chemical and Omics Analyses Provide Insights into Pentlandite Bioleaching by Acidithiobacillus ferriphilus WGS1
Source: Int J Mol Sci. 2026 Jun 26;27(13):5762. doi: 10.3390/ijms27135762 (PMC13362053; doi:10.3390/ijms27135762)
Supplement: Supplementary file 1 [file ijms-27-05762-s001.zip › ijms-4339240-supplementary.pdf]

Supplementary information for:

Integrative Chemical and Omics Analyses Reveal Bioleaching Mechanism of Pentlandite by *Acidithiobacillus ferriphilus* WGS1

Yan Tong [1,2], Yuandong Liu [1,2,*]

[1] School of Minerals Processing and Bioengineering, Central South University, Changsha 410083, China; yantong@csu.edu.cn (Y.T.)
[2] Key Laboratory of Biohydrometallurgy of Ministry of Education, Changsha 410083, China
* Correspondence: yuandong_liu@csu.edu.cn; Tel.: +86-731-88877472

Table S1. Genomic Bioinformatics and Transcriptomics Analysis revealed key genes associated with Pentlandite Bioleaching.

Name	Gene ID	Length(AA)	Log_2FC	Annotation
rus operon				
rusB	GE001588	190	1.52	SignalP_34,TMhelix_1, rusticyanin, Copper-bind (SoxE) domain
rusA	GE001695	188	1.27	SignalP_32,TMhelix_1, rusticyanin
coxD	GE001697	65	1.93	TMhelix_1, cytochrome c oxidase, aa3-type, subunit IV (coxD)
coxC	GE001698	185	1.50	TMhelix_5, cytochrome c oxidase, aa3-type, subunit III (coxC)
coxA	GE001699	628	1.49	TMhelix_14, cytochrome c oxidase, aa3-type, subunit I (coxA)
coxB	GE001700	255	1.74	TMhelix_3, cytochrome c oxidase, aa3-type, subunit II (coxB)
Acop	GE001701	184		TMhelix_1, Acop,conserved hypothetical protein
cyc1	GE001702	230	2.26	SignalP_46,TMhelix_1, cytochrome c552 (cyc1)
cyc2	GE001703	495	2.46	SignalP_31, TMhelix_1, cytochrome c (cyc2
cytochrome o ubiquinol oxidase, bo3 complex				
cyoA	GE000849	322	3.35	TMhelix_4, cytochrome o ubiquinol oxidase, subunit II (cyoA) [1.10.3.-]
cyoB	GE000850	678	2.71	TMhelix_14, cytochrome o ubiquinol oxidase, subunit I (cyoB) [1.10.3.-]

cyoC	GE000851	212	3.32	TMhelix_6, cytochrome o ubiquinol oxidase, subunit III (cyoC) [1.10.3.-]
cyoD	GE000852	119	3.30	TMhelix_3, cytochrome o ubiquinol oxidase, subunit IV (cyoD)
cytochrome o ubiquinol oxidase, bo3 complex				
cyoA	GE000861	311	—	TMhelix_2, cytochrome o ubiquinol oxidase, subunit II (cyoA) [1.10.3.-]
cyoB	GE000862	716	—	TMhelix_15, cytochrome o ubiquinol oxidase, subunit I (cyoB) [1.10.3.-]
cyoC	GE000863	213	—	TMhelix_5, cytochrome o ubiquinol oxidase, subunit III (cyoC) [1.10.3.-]
cyoD	GE000864	129	1.35	TMhelix_3, cytochrome o ubiquinol oxidase, subunit IV (cyoD)
bc1 complex, ubiquinol--cytochrome c reductase				
petC	GE000488	246	1.54	SignalP_22, TMhelix_2, ubiquinol--cytochrome c reductase, cytochrome c1 subunit (petC-1)
petB	GE000489	403	3.28	TMhelix_10, ubiquinol--cytochrome c reductase, cytochrome b subunit (petB-1)
petA	GE000490	207	0.96	TMhelix_1, ubiquinol--cytochrome c reductase, iron-sulfur subunit (petA-1)
SDR1	GE000491	263	—	short-chain dehydrogenase-reductase family
CycA1	GE000492	261	—	cytochrome c4 (cycA-1)
Iro	GE001314	91	1.81	TMhelix_1, iron oxidase (iro) [1.16.3.-]
Iro	GE003013	125	—	SignalP_35,TMhelix_1, iron oxidase (iro) [1.16.3.-]
Hip	GE003113	105	1.91	TMhelix_1, twin-arginine translocation signal domain-containing protein
petC	GE003114	246	1.48	SignalP_22, TMhelix_2, ubiquinol--cytochrome c reductase, cytochrome c1 subunit (petC-2)
petB	GE003115	403	2.88	TMhelix_10, ubiquinol--cytochrome c reductase, cytochrome b subunit (petB-2)
petA	GE003116	207	—	TMhelix_1, ubiquinol--cytochrome c reductase, iron-sulfur subunit (petA-2)
SDR	GE003117	263	—	short-chain dehydrogenase-reductase family
CycA2	GE003118	261	1.4	cytochrome c4 (cycA-2)

Cyc	GE001848	165	—	TMhelix_1, cytochrome c, Cytochrom_C_2 C'
NADH-quinone oxidoreductase				
NuoA	GE000636	119	—	NADH-quinone oxidoreductase subunit A
NuoB	GE000637	166	—	NADH-quinone oxidoreductase subunit NuoB
NuoC	GE000638	202	—	NADH-quinone oxidoreductase subunit NuoC
NuoD	GE000639	417	—	NADH-quinone oxidoreductase subunit NuoD
NuoE	GE000640	163	1.55	NADH-quinone oxidoreductase subunit NuoE
NuoF	GE000641	427	—	NADH-quinone oxidoreductase subunit NuoF
NuoG	GE000642	781	—	NADH-quinone oxidoreductase subunit NuoG
NuoH	GE000643	341	1.18	NADH-quinone oxidoreductase subunit NuoH
NuoI	GE000644	163	—	NADH-quinone oxidoreductase subunit NuoI
NuoJ	GE000645	199	1.24	NADH-quinone oxidoreductase subunit NuoJ
NuoK	GE000646	105	1.95	NADH-quinone oxidoreductase subunit NuoK
NuoL	GE000647	660	—	NADH-quinone oxidoreductase subunit NuoL
NuoM	GE000648	494	—	NADH-quinone oxidoreductase subunit NuoM
NuoN	GE000649	482	—	NADH-quinone oxidoreductase subunit NuoN
atpC	GE003068	141	—	ATP synthase F1, epsilon subunit (atpC)
atpD	GE003069	469	—	ATP synthase F1, beta subunit (atpD)
atpG	GE003070	288	—	ATP synthase F1, gamma subunit (atpG)
atpA	GE003071	514	—	ATP synthase F1, alpha subunit (atpA)
atpH	GE003072	179	—	ATP synthase F1, delta subunit (atpH)
atpF	GE003073	159	1.37	ATP synthase F0, B subunit (atpF)
atpE	GE003074	85	1.53	ATP synthase F0, C subunit (atpE) [3.6.1.14]

atpB	GE003075	248	—	ATP synthase F0, A subunit (atpB) [3.6.3.14]
SBP	GE001238	339	—	SignalP_28, ABC transporter substrate-binding protein, periplasmic solute-binding protein
DoxDA	GE001239	363	—	TMhelix_6, DoxA small subunit DoxD, quinol oxidase;
P21	GE002041	142	—	TMhelix_1, Rhodanese domain
TetH	GE000049	504	2.24	SignalP_22, TMhelix_1, tetrathionate hydrolase
SQR	GE003007	394	1.26	TMhelix_1, pyridine nucleotide-disulfide oxidoreductase
SDO	GE001135	249	—	sulfur dioxygenase,ETHE1, metallo-beta-lactamase family protein, MBL fold metallo-hydrolase
Omp40	GE000453	421	—	SignalP_22,TMhelix_1, major outer membrane protein 40 (omp40)
SOR	GE000410	312	0.97	sulfur oxygenase reductase
SoxA	GE003112	133	—	SignalP_14, L-cysteine S-thiosulfotransferase, sulfur oxidation c-type cytochrome SoxA
SoxY	GE002114	171	4.12	thiosulfate oxidation carrier protein SoxY
SoxZ	GE002115	111	4.00	thiosulfate oxidation carrier complex protein SoxZ
SoxB	GE002117	576	3.64	SignalP_28, thiosulfohydrolase SoxB
RND family genes				
NccA	GE001900	1037	—	TMhelix_12, efflux RND transporter permease subunit, ACR_tran family,The Resistance-Nodulation-Cell Division (RND) Superfamily
NccA	GE002877	1036	—	TMhelix_10, efflux RND transporter permease subunit,ACR_tran family, The Resistance-Nodulation-Cell Division (RND) Superfamily
NccB	GE000083	362	1.05	TMhelix_1, efflux RND transporter periplasmic adaptor subunit,HlyD_3;Biotin_lipoyl_2 domain of CusB or HlyD membrane-fusion;family secretion protein;The Resistance-Nodulation-Cell Division (RND) Superfamily

NccC	GE000084	519	—	TMhelix_1, efflux transporter outer membrane subunit,OEP membrane efflux protein, The Resistance-Nodulation-Cell Division (RND) Superfamily
czcA	GE000757	1037	1.32	TMhelix_12, efflux RND transporter permease subunit,ACR_tran family,The Resistance-Nodulation-Cell Division (RND) Superfamily
czcA	GE002636	1037	—	TMhelix_12, efflux RND transporter permease subunit,ACR_tran family,The Resistance-Nodulation-Cell Division (RND) Superfamily
czcA	GE002853	1037	1.28	TMhelix_12, efflux RND transporter permease subunit,ACR_tran family,The Resistance-Nodulation-Cell Division (RND) Superfamily
NCCB	GE000758	481	—	TMhelix_1, efflux RND transporter periplasmic adaptor subunit,Biotin_lipoyl_2;HlyD_D4 domain of CusB or HlyD membrane-fusion;family secretion protein;like;alpha hairpin domain of cation efflux system protein, CusB, The Resistance-Nodulation-Cell Division (RND) Superfamily
NCCB	GE001799	346	—	TMhelix_1, efflux RND transporter periplasmic adaptor subunit,Biotin_lipoyl_2;HlyD_D4 domain of CusB or HlyD membrane-fusion;family secretion protein;like;alpha hairpin domain of cation efflux system protein, CusB, The Resistance-Nodulation-Cell Division (RND) Superfamily
CNRB	GE002637	479	—	TMhelix_1,efflux RND transporter periplasmic adaptor subunit,HlyD_D23;Biotin_lipoyl_2 domain of CusB or HlyD membrane-fusion;family secretion protein;alpha hairpin domain of cation efflux system protein, CusB;The Resistance-Nodulation-Cell Division (RND) Superfamily
CusC	GE001815	430	1.87	TolC family protein,OEP membrane efflux protein,The Resistance-Nodulation-Cell Division (RND) Superfamily
CusB	GE001816	437	2.48	TMhelix_1,efflux RND transporter periplasmic adaptor subunit,HlyD_D4;Biotin_lipoyl_2 domain

				of CusB or HlyD membrane-fusion;family secretion protein;alpha hairpin domain of cation efflux system protein, CusB;The Resistance-Nodulation-Cell Division (RND) Superfamily
CusA	GE001817	1037	1.57	TMhelix_12,efflux RND transporter permease subunit,ACR_tran;MMPL family;The Resistance-Nodulation-Cell Division (RND) Superfamily
CusF	GE001818	117	—	SignalP_22,secreted,copper-binding protein,CusF_Ec binding periplasmic protein CusF
nccA	GE002043	1098	—	TMhelix_12,efflux RND transporter permease subunit,ACR_tran;MMPL family;The Resistance-Nodulation-Cell Division (RND) Superfamily
CNRC	GE002638	437	—	TolC family protein,OEP membrane efflux protein,The Resistance-Nodulation-Cell Division (RND) Superfamily
P-type ATPases				
CTPD	GE000274	834	—	TMhelix_8, copper-translocating P-type ATPase, E1-E2_ATPase;Hydrolase_3 ATPase;dehalogenase-like hydrolase;domain;dehalogenase-like hydrolase,The P-type ATPase (P-ATPase) Superfamily
CTPD	GE000763	675	—	TMhelix_8, copper-translocating P-type ATPase, E1-E2_ATPase;Hydrolase_3 ATPase;dehalogenase-like hydrolase;domain;dehalogenase-like hydrolase,The P-type ATPase (P-ATPase) Superfamily
CTPD	GE001896	837	1.17	TMhelix_8, copper-translocating P-type ATPase, E1-E2_ATPase;Hydrolase_3 ATPase;dehalogenase-like hydrolase;domain;dehalogenase-like hydrolase,The P-type ATPase (P-ATPase) Superfamily
Q92Z60	GE000896	690	—	TMhelix_7, K(+)-transporting ATPase subunit B, Hydrolase;E1-E2_ATPase dehalogenase-like hydrolase;The P-type ATPase (P-ATPase) Superfamily
Q92Z60	GE001478	828	—	TMhelix_7, K(+)-transporting ATPase subunit B, Hydrolase;E1-E2_ATPase

				dehalogenase-like hydrolase;The P-type ATPase (P-ATPase) Superfamily
CDF family genes				
FIEF	GE000565	388	—	TMhelix_3,cation transporter,Cation_efflux;ZT_dimer efflux family;domain of Zinc Transporter, The Cation Diffusion Facilitator (CDF) Family
dmeF	GE000747	209	—	TMhelix_6,cation transporter,Cation efflux family,The Cation Diffusion Facilitator (CDF) Family
dmeF	GE000750	319	1.42	TMhelix_5,cation transporter,Cation efflux family,The Cation Diffusion Facilitator (CDF) Family
NCCA	GE002627	319	—	TMhelix_5,cation transporter, Cation efflux family,The Cation Diffusion Facilitator (CDF) Family
CorA family genes				
CORA	GE000245	322	—	TMhelix_2, magnesium and cobalt transport protein CorA [Chromatiales bacterium 21-64-14],CorA Mg2+ transporter protein,The CorA Metal Ion Transporter (MIT) Family
CORA	GE003083	310	-1.87	TMhelix_2, magnesium and cobalt transport protein CorA [Chromatiales bacterium 21-64-14],CorA Mg2+ transporter protein,The CorA Metal Ion Transporter (MIT) Family
ABC family genes				
NIKE	GE000095	253	1.10	ABC transporter ATP-binding protein, ABC_tran transporter, The ATP-binding Cassette (ABC) Superfamily
NIKE	GE000210	556	—	ABC transporter ATP-binding protein, ABC_tran transporter, The ATP-binding Cassette (ABC) Superfamily
NIKE	GE000224	314	—	ABC transporter ATP-binding protein, ABC_tran transporter, The ATP-binding Cassette (ABC) Superfamily
NIKE	GE000304	622	—	ABC transporter ATP-binding protein, ABC_tran transporter, The ATP-binding Cassette (ABC) Superfamily
NikA	GE000819	351	—	TMhelix_1, cation ABC transporter substrate-binding protein,ZnuA complex

				component A periplasmic,The ATP-binding Cassette (ABC) Superfamily
FECE	GE000820	275	—	metal ABC transporter ATP-binding protein,ABC_tran;AAA_21transporter;domain, putative AbiEii toxin, Type IV TA system,The ATP-binding Cassette (ABC) Superfamily
NrsRS/OmpR family				
NrsS	GE000217	383	—	hypothetical protein B7Z70_01490,Response_reg;HATPase_c regulator receiver domain;kinase-, DNA gyrase B-, and HSP90-like ATPase, The Uncharacterized Bacterial 5 TMS Protein-1 (UBP1) Family
NrsS	GE000260	421	1.73	hypothetical protein B7Z70_01490,Response_reg;HATPase_c regulator receiver domain;kinase-, DNA gyrase B-, and HSP90-like ATPase, The Uncharacterized Bacterial 5 TMS Protein-1 (UBP1) Family
NrsS	GE000296	362	—	hypothetical protein B7Z70_01490,Response_reg;HATPase_c regulator receiver domain;kinase-, DNA gyrase B-, and HSP90-like ATPase, The Uncharacterized Bacterial 5 TMS Protein-1 (UBP1) Family
NrsS	GE000552	495	—	hypothetical protein B7Z70_01490,Response_reg;HATPase_c regulator receiver domain;kinase-, DNA gyrase B-, and HSP90-like ATPase, The Uncharacterized Bacterial 5 TMS Protein-1 (UBP1) Family
OmpR	GE000034	469	—	sigma-54-dependent Fis family transcriptional regulator, partial,Sigma54_activat;Response_reg;HTH_8;interaction domain;regulator receiver domain;regulatory protein, Fis family;domain (dynein-related subfamily),The Type VI Symbiosis/Virulence Secretory System (T6SS) Family
OmpR	GE000085	249	1.30	sigma-54-dependent Fis family transcriptional regulator, partial,Sigma54_activat;Response_reg;HTH_8;interaction domain;regulator receiver domain;regulatory protein, Fis

				family;domain (dynein-related subfamily),The Type VI Symbiosis/Virulence Secretory System (T6SS) Family
OmpR	GE000151	275	—	sigma-54-dependent Fis family transcriptional regulator, partial,Sigma54_activat;Response_reg;HTH_8;interaction domain;regulator receiver domain;regulatory protein, Fis family;domain (dynein-related subfamily),The Type VI Symbiosis/Virulence Secretory System (T6SS) Family
OmpR	GE000218	121	—	sigma-54-dependent Fis family transcriptional regulator, partial,Sigma54_activat;Response_reg;HTH_8;interaction domain;regulator receiver domain;regulatory protein, Fis family;domain (dynein-related subfamily),The Type VI Symbiosis/Virulence Secretory System (T6SS) Family
OmpR	GE000219	359	—	sigma-54-dependent Fis family transcriptional regulator, partial,Sigma54_activat;Response_reg;HTH_8;interaction domain;regulator receiver domain;regulatory protein, Fis family;domain (dynein-related subfamily),The Type VI Symbiosis/Virulence Secretory System (T6SS) Family
NCCH	GE001616	209	—	RNA polymerase sigma factor RpoE,Sigma70_ECF region 4;rThe Type 9 Secretory System (T9SS) Family
ArsR-SmtB family				
NMTR	GE000735	138	1.27	MULTISPECIES: ArsR family transcriptional regulator;HTH_20 regulatory protein, arsR family;
NMTR	GE002874	138	1.98	MULTISPECIES: ArsR family transcriptional regulator;HTH_20 regulatory protein, arsR family;
NMTR	GE003011	134	—	MULTISPECIES: ArsR family transcriptional regulator;HTH_20 regulatory protein, arsR family;

CMTR	GE001130	120	—	ArsR family transcriptional regulator,HTH_5 domain;regulatory protein, arsR family
CMTR	GE001370	121	—	ArsR family transcriptional regulator,HTH_5 domain;regulatory protein, arsR family
KMTR	GE001195	119	1.42	transcriptional regulator,HTH_20;MarR_2;HTH_IclR domain;regulatory protein, arsR family;family;helix-turn-helix domain
Other Ni-homeostasis proteins				
cnrT	GE001812	350	—	TMhelix_9,DMT family transporter,EamA transporter family, The Drug/Metabolite Transporter (DMT) Superfamily
nreB	GE003005	413	—	TMhelix_11, MFS transporter,MFS_1_like;LacY_symp Facilitator Superfamily;secretion effector;like family;proton/sugar symporter,The Major Facilitator Superfamily (MFS)
NCCN	GE003087	190	—	TMhelix_4, isoprenylcysteine carboxylmethyltransferase family protein,ICMT;PEMT;ERG4_ERG24 carboxyl methyltransferase (ICMT) family;methyltransferase;The Putative Integral Membrane Steroid reductase Family

Note: A dash ("—") in the Log_2FC column does not mean that the corresponding gene is absent in WGS1. Instead, it indicates that no effective transcription signal of this gene was detected in the current transcriptome data, so the fold-change in differential expression could not be calculated.
